# Transformation of Thia[7]helicene to Aza[7]helicenes and [7]Helicene-like Compounds via Aromatic Metamorphosis

**DOI:** 10.3390/molecules27030606

**Published:** 2022-01-18

**Authors:** Keisuke Uematsu, Chikara Hayasaka, Ko Takase, Keiichi Noguchi, Koji Nakano

**Affiliations:** 1 Department of Applied Chemistry, Graduate School of Engineering, Tokyo University of Agriculture and Technology, 2-24-16 Naka-cho, Koganei 184-8588, Japan; Uematsu.Keisuke@technopro.com (K.U.); s212228t@st.go.tuat.ac.jp (C.H.); Ko.Takase@mitsuichemicals.com (K.T.); 2 Instrumentation Analysis Center, Tokyo University of Agriculture and Technology, 2-24-16 Naka-cho, Koganei 184-8588, Japan; knoguchi@cc.tuat.ac.jp

**Keywords:** [*n*]helicenes, aromatic metamorphosis, desulfurative lithiation, nucleophilic aromatic substitution, thiophene, silole, phosphole, pyrrole, circular dichroism, circularly polarized luminescence

## Abstract

[*n*]Helicenes with helically twisted structures have attracted increasing interest owing to their unique properties. Therefore, it has been an important issue to develop facile synthetic methodologies which allow access to a variety of [*n*]helicenes. Here we report the synthesis of [7]helicenes and [7]helicene-like compounds from the thia[7]helicene as a common starting material. Desulfurative dilithiation of the thia[7]helicene and the subsequent reaction with silicon and phosphorus electrophiles afforded the silole- and phosphole-fused [7]helicene-like compounds, respectively. The cyclopentadiene-fused [7]helicene-like compound and the pyrrole-fused aza[7]helicenes were also successfully synthesized via twofold S_N_Ar reactions of the thia[7]helicene *S,S*-dioxide with the carbon and nitrogen nucleophiles, respectively. The thia[7]helicene *S*,*S*-dioxide showed a slightly red-shifted absorption spectrum than the parent thia[7]helicene, which was well demonstrated by the theoretical calculations. The substituents on the silicon atom of silole-fused [7]helicene-like compounds have little impact on the longest absorption maximum. Such little effect of the substituents on absorption properties was also observed for cyclopentadiene-fused [7]helicene-like compounds and aza[7]helicenes and was well demonstrated by the theoretical calculations. The thia[7]helicene *S*,*S*-dioxide and the silole-fused [7]helicene-like compound exhibited bright blue emission, and the cyclopentadiene-fused [7]helicene-like compound and the aza[7]helicenes showed strong violet emission. Each single enantiomer of the aza[7]helicenes showed circularly-polarized luminescence with the dissymmetry factors of 4.2~4.4 × 10^−3^.

## 1. Introduction

[*n*]Helicenes are *ortho*-fused polycyclic aromatic compounds in which all rings are angularly arranged to form screw-shaped helical structures [1,2,3]. Those composed of six or more benzene rings exhibit stable helical chirality owing to an intramolecular steric repulsion. The π-extended, nonplanar, and chiral structures of [*n*]helicenes endow them with unique properties and offer promising applications in various fields such as nonlinear optics [4], organic semiconductors [5,6], organic light-emitting materials [7], catalysts [8,9], and circularly polarized luminescent materials [10]. The prototypical [*n*]helicene is carbohelicenes whose aromatic rings consist of only carbon atoms. Another class of [*n*]helicenes is heterohelicenes with one or more heteroaromatic rings, such as pyrrole, furan, thiophene, and pyridine, in the helical skeletons. Besides these [*n*]helicenes composed of only aromatic rings, helicene-like compounds with non-aromatic rings, such as silole and phosphole, in the helical skeletons have also been developed. Heterohelicenes and helicene-like compounds have received growing interest because the introduction of heteroatom(s) into the π-conjugated framework is effective for controlling the electronic structure of the π-conjugated molecules and providing unique functions.

A variety of synthetic methodologies for helicenes have been developed. Photocyclization of the stilbene skeleton has been a conventional and common one [11]. Diels–Alder reaction [12], Friedel–Crafts-type reaction [5,13], transition metal-catalyzed [2 + 2 + 2] cycloaddition [14,15,16,17,18], cross-coupling reaction [19], and olefin metathesis [20,21] have been found to be effective for helicene synthesis during the last decades. These synthetic methodologies have undoubtedly contributed to a great progress in helicene chemistry. However, they often require lengthy multistep synthesis, which would be disadvantageous for structural diversity of helicenes. Therefore, there is still a tremendous need for developing more diversity-oriented synthetic methodologies.

Recently, it has been reported that dibenzothiophenes can be converted into a variety of ring systems, such as dibenzosiloles, dibenzophospholes, fluorenes, and carbazoles [22,23,24,25,26]. Transformation to dibenzosiloles and dibenzophospholes involves (1) the generation of 1,1′-dianions via desulfurative dilithiation with lithium metal and (2) the subsequent reaction of the resulting 1,1′-dianions with silicon and phosphorus electrophiles, respectively. On the other hand, dibenzothiophenes are converted into fluorenes and carbazoles via (1) oxidation to *S*,*S*-dioxides and (2) the consecutive inter- and intramolecular S_N_Ar reactions with carbon and nitrogen nucleophiles. This methodology named as “aromatic metamorphosis” would be a powerful tool for synthesizing π-extended compounds since a wide range of compounds containing the dibenzothiophene unit have been reported. Indeed, [8]circulenes [27,28] and spiro π-conjugated compounds [29] have been prepared by using this methodology. Yorimitsu and co-workers have also reported the transformation of dithia[8]helicene to its carbazole and fluorene analogs [30].

**Figure 1:**
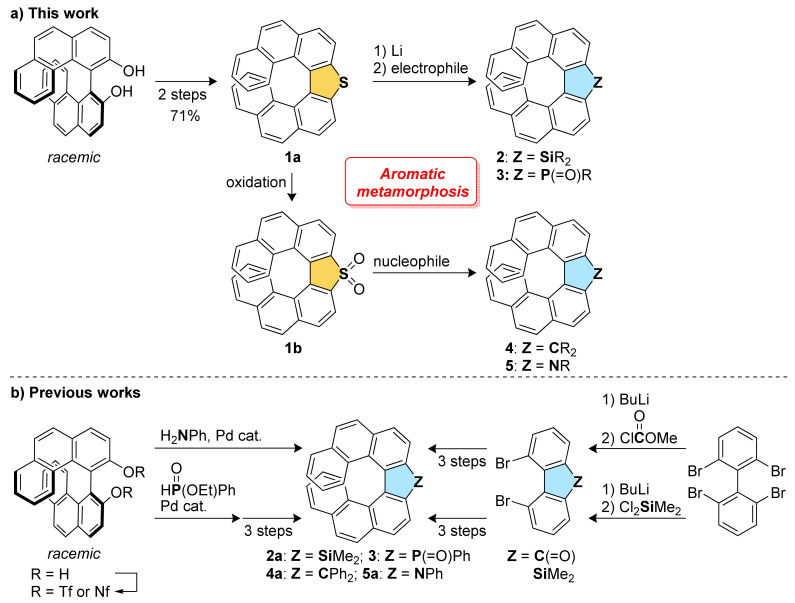
Synthetic routes to [7]helicenes and [7]helicene-like compounds.

In this context, we envisaged that a series of silole-, phosphole-, and cyclopentadiene-fused [7]helicene-like compounds **2**–**4** and pyrrole-fused aza[7]helicenes **5** can be synthesized from a single thia[7]helicene, diphenanthro[3,4-*b*:4′,3′-*d*]thiophene (**1a**) [31,32] as a common starting material (Figure 1a). We have previously reported the synthesis of **2a**, **3**, **4a**, and **5a** (Figure 1b) [33,34,35,36]. Each heteroatom and the bridging carbon was introduced at the early stage in the synthetic sequence. In addition, all the synthetic routes require costly transition metal catalysts. In contrast, the synthetic routes in this work are transition metal-free and can provide a series of [7]helicene-like compounds and aza[7]helicenes via late-stage diversification of thia[7]helicene **1a**, therefore achieving more diversity-oriented synthesis. Aromatic metamorphosis of the dibenzothiophene unit to carbazole and fluorene units has been applied to the helicene synthesis as described above [30], but its application to silole- and phosphole-fused helicene-like compounds has never been demonstrated. Herein we report the synthesis of a series of [7]helicene-like compounds and aza[7]helicenes through aromatic metamorphosis. Their photophysical properties are also discussed through comparison with the previously reported analogs.

## 2. Results and Discussion

**Scheme 1:**
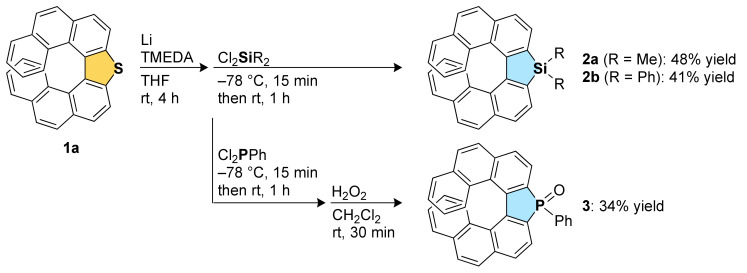
Synthesis of silole- and phosphole oxide-fused [7]helicene-like compounds.

Aromatic metamorphosis of thia[7]helicene **1a** to its silole and phosphole oxide analogs **2** and **3**, respectively, was first investigated (Scheme 1). The desulfurative dilithiation of **1a** with lithium metal followed by the trapping with MeI has been examined previously [31]. In the literature, the desired methylated compound was not obtained, and the main product was found to be the reduced compound, 4,4′-biphenanthrene. On the other hand, we have recently reported that the dianion formed by the reaction of 3,3′-dibromo-4,4′-biphenanthrene with BuLi can react with SiCl_4_ to give the double [7]helicene-like compound with a silicon spiro center [37]. Encouraged by the recent progress in aromatic metamorphosis of dibenzothiophenes [26] and our recent result, we conducted the reaction of thia[7]helicene **1a** with 5 equivalents of lithium metal in the presence of 2 equivalents of TMEDA in THF at room temperature, which is the optimized conditions in the recent report on aromatic metamorphosis (Scheme 1). To our delight, the subsequent reaction with commercially available Cl_2_SiMe_2_ successfully afforded the silole-fused [7]helicene-like compound **2a** in 48% yield. In addition, the reaction with Cl_2_SiPh_2_ proceeded to give the new silole-fused [7]helicene-like compound **2b** in 41% yield. Phosphorous electrophile was also found to be applicable, and the phosphole oxide-fused [7]helicene-like compound **3** was obtained through the reaction of the dianion with Cl_2_PPh and the following oxidation.

**Scheme 2:**
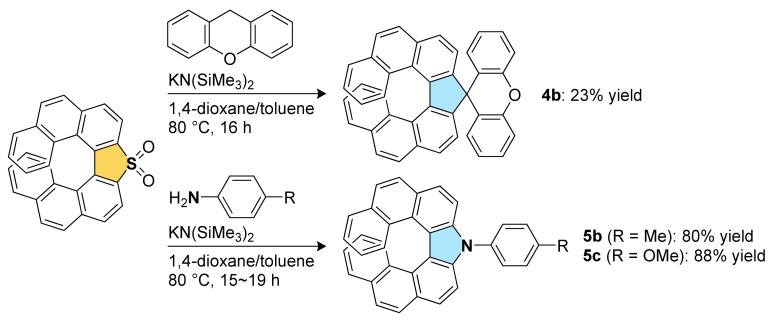
Synthesis of the cyclopentadiene-fused [7]helicene-like compound and pyrrole-fused aza[7]helicenes.

Transformation of thia[7]helicene *S*,*S*-dioxide **1b**, which can be easily prepared from **1a** by oxidation, was also examined (Scheme 2). According to the literature in which dibenzothiophene *S*,*S*-dioxides are converted into fluorenes [24], the reaction of **1b** with xanthene, a commercially available carbon nucleophile, was conducted in the presence of KN(SiMe_3_)_2_ as a base. Although the yield is unexpectedly low, the desired spirocyclic carbo[7]helicene-like compound **4b** was obtained (23% yield). Next, the transformation of **1b** to aza[7]helicenes **5** was investigated. Arylamines with electron-donating groups, such as *p*-toluidine and *p*-anisidine, reacted with **1b** smoothly, affording the corresponding aza[7]helicenes **5b** and **5c**, respectively, in high yields (80% for **5b** and 88% for **5c**). The reaction with electron-deficient arylamines, such as *p*-(trifluoromethyl)aniline and 4-aminobenzonitrile, resulted in no production of the aza[7]helicenes, which would be due to low nucleophilicity of these arylamines [22].

**Figure 2:**
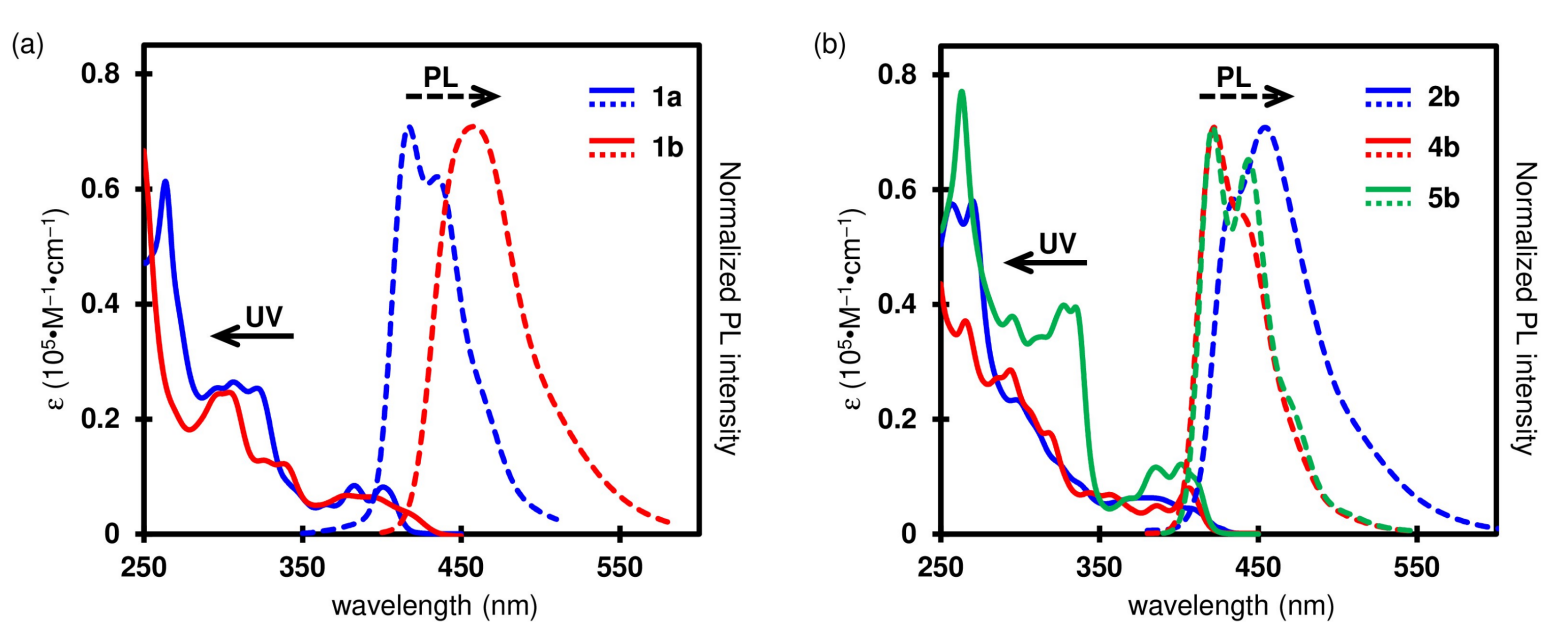
UV−vis absorption (solid line) and PL (broken line) spectra of (**a**) **1a** and **1b** in CH_2_Cl_2_ (1.0 × 10^−5^ M) and (**b**) **2b**, **4b**, and **5b** in CH_2_Cl_2_ (1.0 × 10^−5^ M). The spectra of **5c** are omitted since they are almost the same as those of **5b**.

The UV–vis absorption and photoluminescence (PL) spectra of new silole- and cyclopentadiene-fused [7]helicene-like compounds **2b** and **4b** and pyrrole-fused aza[7]helicenes **5b** and **5c** were measured to evaluate their photophysical properties (Figure 2). Thiophene-fused thia[7]helicene **1a** and its *S*,*S*-dioxide **1b** are known compounds, but only the absorption property of **1a** in cyclohexane has been reported. Therefore, their absorption and fluorescence spectra in CH_2_Cl_2_ were also measured. The photophysical data are summarized in Table 1, together with those of the previously reported **2a**, **4a**, and **5a** for comparison. We also performed theoretical calculations by density functional theory (DFT) and time-dependent (TD) DFT methods at the B3LYP/6-31G(d) level of theory to understand the experimental photophysical properties.

**Figure 3:**
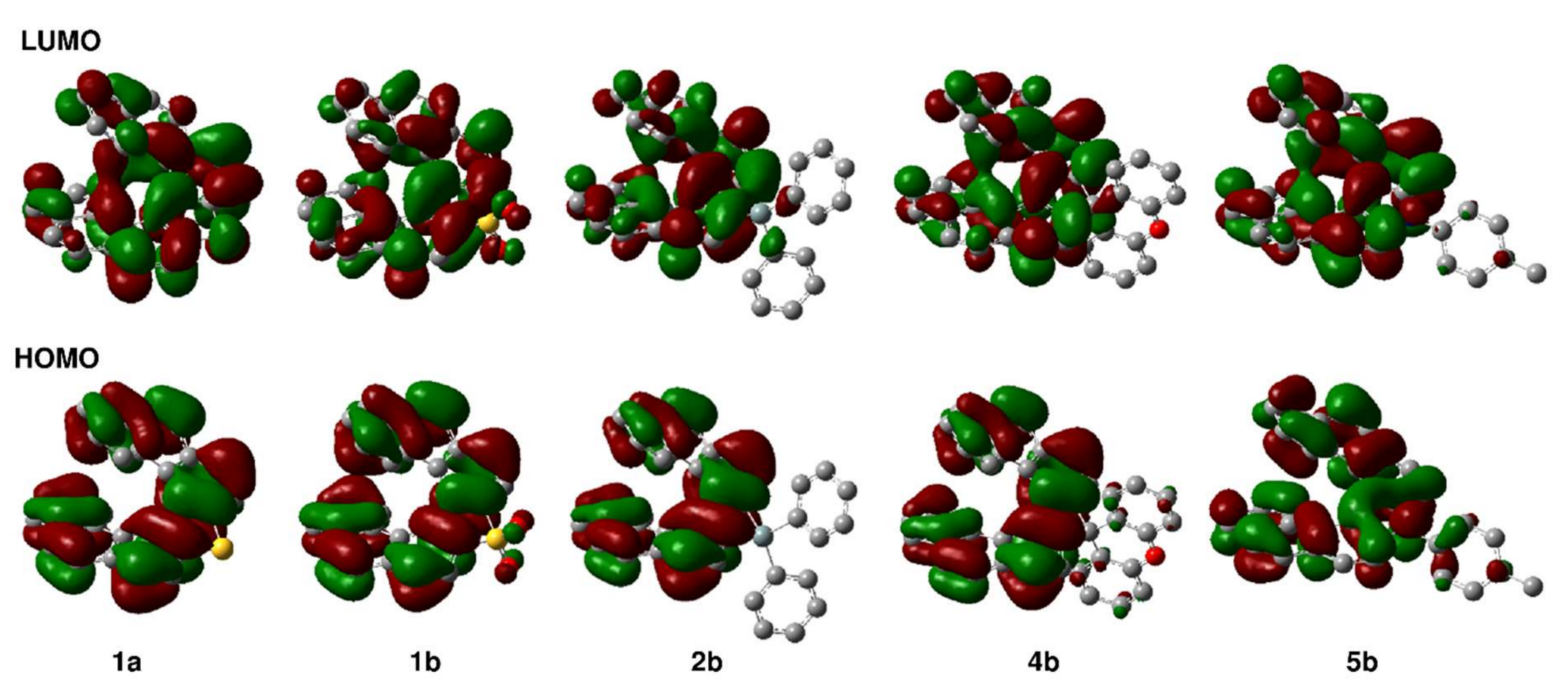
Frontier molecular orbitals of **1a**, **1b**, **2b**, **4b**, and **5b** calculated by DFT method at the B3LYP/6-31G(d) level of theory.

Thia[7]helicene **1a** showed the longest absorption maximum (λ_abs_) at 401 nm and an emission maximum (λ_em_) at 417 nm with a shoulder at 435 nm (Figure 2a). Its fluorescence intensity is weak, and the absolute quantum yield (ϕ) was estimated to be <1%. The *S*,*S*-dioxide compound **1b** gave a slightly red-shifted absorption band with a shoulder at 415 nm. The emission maximum (458 nm) is significantly red-shifted with a higher absolute quantum yield (4%), compared to those of **1a**, exhibiting a bright blue emission. The TD–DFT calculations demonstrated a slightly red-shifted absorption characteristic of **1b** (Table S11 in the supplementary material). The longest absorption wavelengths of **1a** and **1b** were estimated to be 387 nm and 406 nm, respectively. The energy levels of the HOMO and HOMO–1 of **1a** are almost identical (−5.42 eV and −5.45 eV), and the HOMO–1 → LUMO transition mainly contributes to the longest-wavelength absorption band of **1a**. On the other hand, the HOMO → LUMO transition is the main contribution to the longest-wavelength absorption band of **1b**. The HOMOs of both compounds are delocalized over the entire skeleton, but without any contribution from the sulfur atoms (Figure 3). The HOMO energy level of **1b** is 0.40 eV lower than that of **1a** owing to the electron-withdrawing effect of the *S*,*S*-dioxide moiety. The LUMOs are delocalized over the entire skeleton including the sulfur atoms. It is notable that the difference between LUMO energy levels (0.62 eV) is larger than that between the HOMO energy levels. It may be due to the effective σ*–π* conjugation between the exocyclic S–O σ* orbitals and the endocyclic butadiene π* orbital of the thiophene unit [38], effectively lowering the LUMO energy level of **1b**.

The silole-fused [7]helicene-like compound **2b** exhibited a relatively broad absorption spectrum with a shoulder at 411 nm and an absorption edge at 434 nm, which are almost the same as those of the dimethyl analog **2a** (λ_abs_: 412 nm; absorption edge: 431 nm) [35]. The absorption wavelengths estimated by TD–DFT calculation (392 nm for **2a**; 396 nm for **2b**) agree with these experimental absorption characteristics (Table S11 in supplementary material). The features of a silole are well reflected in the LUMO of **2b**, as observed for **2a**. The LUMO is distributed over the silicon atom owing to the σ*–π* conjugation between Si–C(phenyl) σ* orbitals and the butadiene π* orbital of the silole unit (Figure 3). Except for this effect, the phenyl substituents of **2b** have no obvious contribution in the frontier orbitals. Therefore, the molecular orbital distributions in the HOMOs and LUMOs of **2a** and **2b** are almost the same as each other, giving almost the same λ_abs_. A bright blue emission was observed for **2b** (λ_em_: 454 nm), while the absolute quantum yield of **2b** (11%) is much lower than that of **2a** (23%).

Photophysical properties of the cyclopentadiene-fused [7]helicene-like compound **4b** and the pyrrole-fused aza[7]helicenes **5b** and **5c** were also found to be similar to those of their analogs **4a** and **5a**, respectively. All compounds exhibit strong violet emission at 421–422 nm. As indicated by theoretical calculations (Table S11 in supplementary material), the bridging of the two phenyl groups in **4a** by an oxygen atom and the introduction of the electron-donating groups at para-position of *N*-phenyl group in **5a** have little impact on their electronic structure. The compound **4b** contains a spirocyclic structure, but a through-space orbital interaction known as spiroconjugation [39,40,41] between the 4,4′-biphenanthrene and diphenyl ether units is not observed.

**Figure 4:**
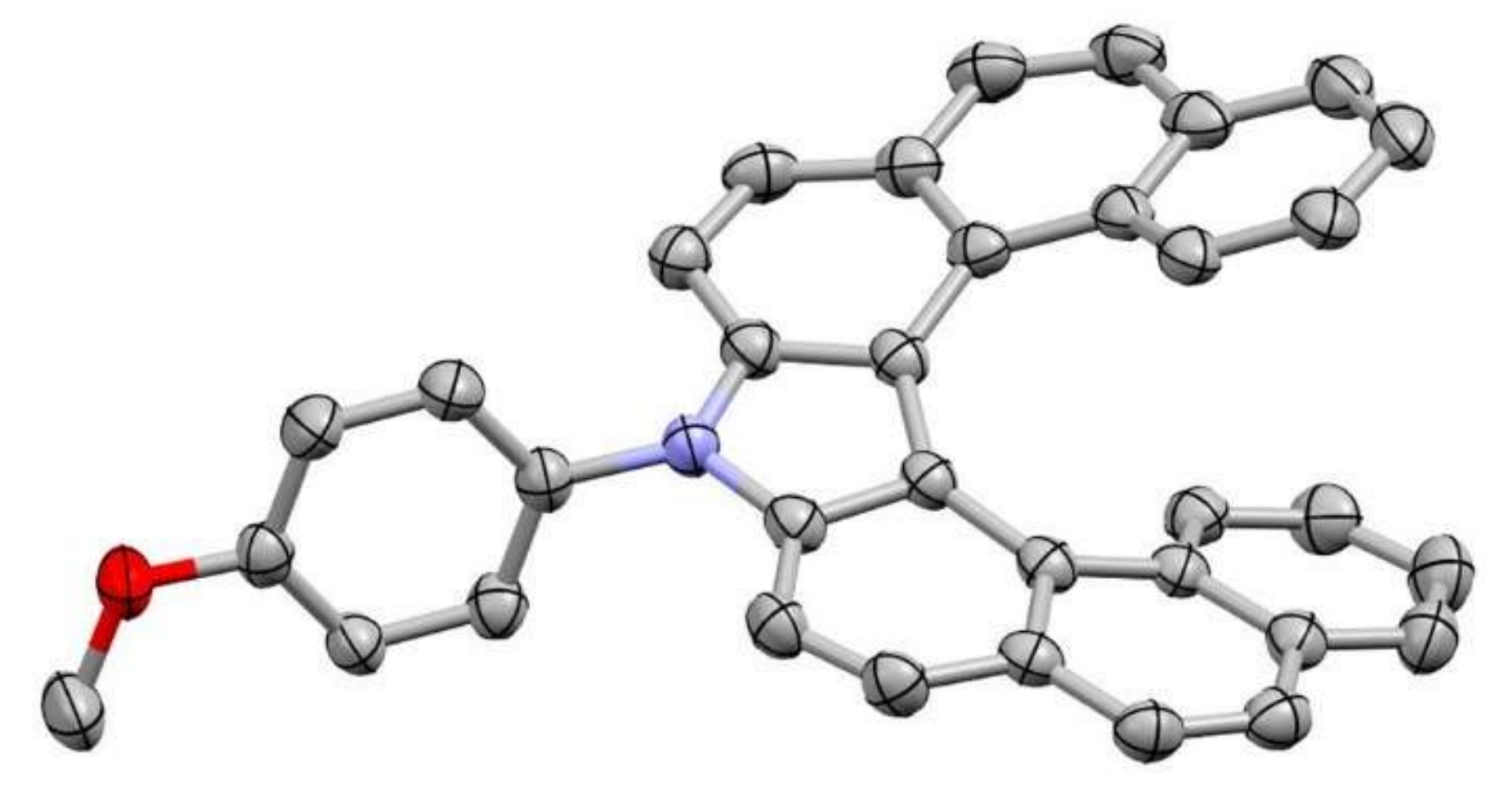
ORTEP drawing of (*M*)-**5c** with thermal ellipsoids at 50% probability. All hydrogen atoms and the crystal solvent molecule (CHCl_3_) are omitted for clarity.

**Figure 5:**
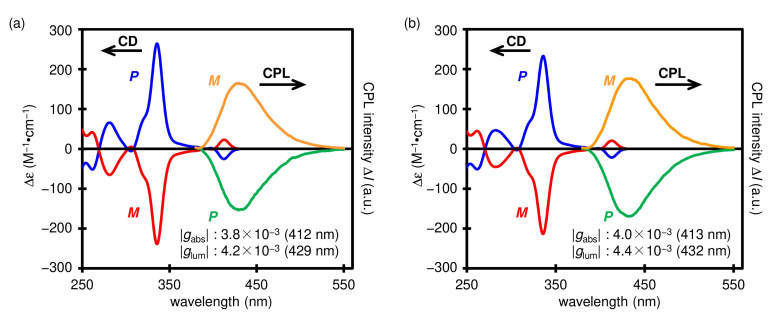
CD (in CH_2_Cl_2_) and CPL (in 2-Me-THF) spectra of (**a**) **5b** and (**b**) **5c**. The *g*_abs_ and *g*_lum_ values were calculated at the longest CD maximum and the CPL maximum, respectively. Wavelength for the calculated values in parentheses.

Fortunately, we found the optical resolution conditions for aza[7]helicenes **5b** and **5c** by using chiral HPLC. In addition, the absolute configuration of the obtained single enantiomer of **5c** was unambiguously identified by single-crystal X-ray analysis (Figure 4). The chiroptical properties of **5b** and **5c** were evaluated by CD and CPL spectroscopies (Figure 5). Both **5b** and **5c** showed mirror-image CD spectra with a Cotton effect in the longest absorption region of their absorption spectra. The (*P*)-isomer of **5c** showed a negative Cotton effect at its longest absorption band along with two positive Cotton effects at around 340 nm and 280 nm. Such a characteristic of the CD spectrum of (*P*)-**5c** is identical to that of (*P*)-**5a** we have previously reported [33]. Based on these facts, we assigned the absolute configuration of the single enantiomer of **5b**, which showed a negative Cotton effect at around 340 nm, to be *P*. The dissymmetry factors in absorption (*g*_abs_) of (*P*)-**5b** and (*P*)-**5c** were estimated to be −3.8 × 10^−3^ and −4.0 × 10^−3^, respectively, at the maximum of the first Cotton effects. Enantiomers of **5b** and **5c** exhibited clear CPL signals. The CPL sign from (*P*)-enantiomers of them is negative. The dissymmetry factors in emission (*g*_lum_) of (*P*)-**5b** and (*P*)-**5c** were −4.2 × 10^−3^ (429 nm) and −4.4 × 10^−3^ (432 nm), respectively, which are comparable to those of the previously reported chiral small organic molecules [42].

## 3. Materials and Methods

### 3.1. General Procedures

All manipulations involving air- and/or moisture-sensitive compounds were carried out with the standard Schlenk technique under argon. Analytical thin-layer chromatography was performed on a glass plate coated with 0.25-mm 230–400-mesh silica gel containing a fluorescent indicator. Column chromatography was performed by using silica gel (spherical neutral, particle size: 63–210 μm). The recycling preparative HPLC was performed with YMC-GPC T-2000 and T-4000 columns (chloroform as an eluent) (YMC Co., Ltd., Kyoto, Japan). Most of the reagents were purchased from commercial suppliers, such as Sigma-Aldrich Co. LLC (St. Louis, MO, USA), Tokyo Chemical Industry Co., Ltd. (Tokyo, Japan), and Kanto Chemical Co., Inc. (Tokyo, Japan), and used without further purification unless otherwise specified. Commercially available anhydrous solvents were used for air- and/or moisture sensitive reactions. Diphenanthro[3,4-*b*:4′,3′-*d*]thiophene (**1a**) and diphenanthro[3,4-*b*:4′,3′-*d*]thiophene *S*,*S*-dioxide (**1b**) were prepared according to the literature [31].

NMR spectra were recorded in CDCl_3_ on a JEOL−ECX400 spectrometer (^1^H 400 MHz; ^13^C 101 MHz) or JEOL−ECA500 spectrometer (^1^H 500 MHz; ^13^C 126 MHz) (JEOL Ltd., Tokyo, Japan). Chemical shifts are reported in ppm relative to the internal standard signal (0 ppm for Me_4_Si in CDCl_3_) for ^1^H and the deuterated solvent signal (77.16 ppm for CDCl_3_) for ^13^C. Data are presented as follows: chemical shift, multiplicity (s = singlet, d = doublet, t = triplet, m = multiplet and/or multiple resonances), coupling constant in hertz (Hz), and signal area integration in natural numbers. High resolution mass spectra are taken with a Bruker Daltonics micrOTOF-QII mass spectrometer (Bremen, Germany) by atmospheric pressure chemical ionization-time-of-flight (APCI–TOF) method. UV–Vis absorption spectra were recorded on a JASCO V–650 spectrophotometer (JASCO Corporation, Tokyo, Japan). Photoluminescence spectra were recorded on a JASCO FP−6500 spectrofluorometer (JASCO Corporation, Tokyo, Japan). Absolute quantum yields were determined by absolute quantum yield measurement system with a JASCO ILF–533 integrating sphere (JASCO Corporation, Tokyo, Japan). HPLC analyses and optical resolution were carried out using a DAICEL CHIRALPAK^®^ IA-3 column (Daicel Corporation, Tokyo, Japan) (4.6 mm × 250 mm) and a DAICEL CHIRALPAK^®^ IA column (Daicel Corporation, Tokyo, Japan) (20 mm × 250 mm), respectively. Circular dichroism (CD) spectra were recorded on a JASCO J–725 spectrometer (JASCO Corporation, Tokyo, Japan). CPL spectra were measured by using a JASCO CPL–300 spectrometer (JASCO Corporation, Tokyo, Japan).

### 3.2. Synthesis

#### 3.2.1. 9,9-Dimethyl-9*H*-diphenanthro[3,4-*b*:4′,3′-*d*]silole (**2a**)

A 20-mL Schlenk tube was charged with lithium (7 mg, 1.0 mmol), THF (1.0 mL), *N*,*N*,*N*′,*N*′-tetramethylethylenediamine (TMEDA) (60 μL, 0.40 mmol) under argon atmosphere. After the mixture was stirred at room temperature for 10 min, diphenanthro[3,4-*b*:4′,3′-*d*]thiophene (**1a**) (77 mg, 0.20 mmol) was added. The resulting mixture was stirred vigorously at room temperature for 4 h, and then cooled to −78 °C. After adding Cl_2_SiMe_2_ (48 μL, 0.40 mmol) under argon atmosphere, the resulting mixture was stirred at −78 °C for 15 min and at room temperature for an additional 1 h. The reaction was quenched with water at 0 °C, and the resulting mixture was extracted with AcOEt (10 mL × 3). The combined organic layers were washed with brine, dried over Na_2_SO_4_, filtered, and concentrated under reduced pressure. The crude residue was purified by silica-gel column chromatography (hexane as an eluent) and then by recycling preparative HPLC to give the title compound **2a** as a yellow solid (39 mg, 48% yield). The ^1^H NMR data were identical to that reported in the literature [35].

#### 3.2.2. 9,9-Diphenyl-9*H*-diphenanthro[3,4-*b*:4′,3′-*d*]silole (**2b**)

The crude product was obtained by using lithium (14 mg, 2.0 mmol), THF (4 mL), TMEDA (0.12 mL, 0.80 mmol), diphenanthro[3,4-*b*:4′,3′-*d*]thiophene (**1a**) (154 mg, 0.40 mmol), and Cl_2_SiPh_2_ (0.17 mL, 0.80 mmol) at room temperature for 19 h. Purification by silica-gel column chromatography (CH_2_Cl_2_/hexane = 7/3 as an eluent; *R*_f_ = 0.82) and then by recycling preparative HPLC gave the title compound **2b** as a yellow solid (88 mg, 41% yield): ^1^H NMR (400 MHz, CDCl_3_) δ 7.95 (d, *J* = 7.3 Hz, 2H), 7.79 (d, *J* = 7.3, 2H), 7.77–7.75 (m, 4H), 7.65 (d, *J* = 8.7 Hz, 2H), 7.52 (d, *J* = 8.7 Hz, 2H), 7.43–7.35 (m, 8H), 7.32 (d, *J* = 7.8 Hz, 2H), 6.94 (t, *J* = 7.8 Hz, 2H), 6.37 (t, *J* = 8.2 Hz, 2H); ^13^C NMR (101 MHz, CDCl_3_) δ 149.0, 136.6, 135.8, 134.6, 132.8, 132.1, 130.4, 130.3, 130.0, 129.1, 128.4, 128.1, 127.6, 126.7, 126.5, 126.2, 125.1, 123.1; HRMS–APCI^+^ (*m*/*z*) calcd for C_40_H_27_Si^+^ ([M+H]^+^) 535.1877, found 535.1876.

#### 3.2.3. 9-Phenylnaphtho[1,2-*e*]phenanthro[3,4-*b*]phosphindole 9-oxide (**3**)

A 20-mL Schlenk tube was charged with lithium (7 mg, 1.0 mmol), THF (2 mL), *N*,*N*,*N*′,*N*′-tetramethylethylenediamine (TMEDA) (60 μL, 0.40 mmol) under argon atmosphere. After the mixture was stirred at room temperature for 10 min, diphenanthro[3,4-*b*:4′,3′-*d*]thiophene (**1a**) (77 mg, 0.20 mmol) was added. The resulting mixture was stirred vigorously at room temperature for 4 h, and then cooled to −78 °C. After adding Cl_2_PPh (54 μL, 0.40 mmol) under argon atmosphere, the resulting mixture was stirred at −78 °C for 10 min and at room temperature for an additional 30 min. The reaction mixture was concentrated under reduced pressure. The resulting residue was dissolved with CH_2_Cl_2_ (10 mL) and passed through a short pad of neutral alumina under argon atmosphere. The alumina pad was washed with CH_2_Cl_2_ (5 mL), and the combined filtrates were concentrated under reduced pressure. The resulting residue was dissolved in CH_2_Cl_2_ (5 mL), and H_2_O_2_ (0.40 mL, 35% aqueous solution) was added in one portion. After the resulting mixture was stirred at room temperature for 30 min, saturated aqueous Na_2_S_2_O_3_ (5 mL) was added slowly to the reaction mixture. The organic layer was separated, and the aqueous layer was extracted with AcOEt (10 mL × 2). The combined organic layers were dried over Na_2_SO_4_, filtered, and concentrated under reduced pressure. The resulting residue was purified by silica-gel column chromatography (CH_2_Cl_2_/AcOEt = 4/1 as an eluent, *R*_f_ = 0.25) and then by recycling preparative HPLC to give the title compound **3** as a yellow solid (32 mg, 34% yield). The ^1^H NMR data were identical to that reported in the literature [34].

#### 3.2.4. Spiro[cyclopenta[1,2-*c*:4,3-*c*′]diphenanthrene-9,9′-xanthene] (**4b**)

A 20-mL Schlenk tube was charged with diphenanthro[3,4-*b*:4′,3′-*d*]thiophene *S*,*S*-dioxide (**1b**) (50 mg, 0.12 mmol), xanthene (27 mg, 0.18 mmol), and 1,4-dioxane (2.5 mL) under argon atmosphere. A solution of KN(SiMe_3_)_2_ (0.5 M in toluene, 0.60 mL, 0.30 mmol) was added to the mixture at an ambient temperature. The resulting solution was stirred at 80 °C for 16 h. After the reaction was quenched with saturated aqueous NH_4_Cl (3 mL), the resulting mixture was extracted with CH_2_Cl_2_ (10 mL × 3). The combined organic layers were dried over Na_2_SO_4_, filtered, and concentrated under reduced pressure. The crude residue was dissolved in CH_2_Cl_2_ and passed through a short pad of silica gel. The filtrate was concentrated, and the resulting crude residue was purified by recycling preparative HPLC to give the title compound **4b** as a pale-yellow solid (15 mg, 23% yield): ^1^H NMR (500 MHz, CDCl_3_) *δ* 7.81 (d, *J* = 7.5 Hz, 2H), 7.76–7.71 (m, 6H) 7.65 (d, *J* = 8.0 Hz, 2H), 7.47 (d, *J* = 8.0 Hz, 2H), 7.30 (dd, *J* = 8.0, 1.2 Hz, 2H), 7.23–7.20 (m, 2H), 7.14 (t, *J* = 7.5 Hz, 2H), 6.73–6.70 (m, 2H), 6.52 (dd, *J* = 7.7, 1.2 Hz, 2H), 6.36–6.33 (m, 2H); ^13^C NMR (101 MHz, CDCl_3_) 156.3, 152.0, 136.7, 132.6, 131.8, 131.0, 129.6, 128.5, 128.27, 127.7, 127.6, 127.0, 126.9, 126.8, 126.6, 123.7, 123.5, 123.4, 117.2, 54.49; HRMS–APCI^+^ (*m/z*) calcd for C_41_H_25_O^+^ ([M+H]^+^) 533.1900, found 533.1900.

#### 3.2.5. 9-(4-Methylphenyl)-9*H*-dinaphtho[2,1-*c*:1′,2′-*g*]carbazole (**5b**)

A 20-mL Schlenk tube was charged with diphenanthro[3,4-*b*:4′,3′-*d*]thiophene *S*,*S*-dioxide (**1b**) (63 mg, 0.15 mmol), *p*-toluidine (32 mg, 0.30 mmol), and 1,4-dioxane (2.5 mL) under argon atmosphere. A solution of KN(SiMe_3_)_2_ (0.5 M in toluene, 0.90 mL, 0.45 mmol) was added to the mixture at an ambient temperature. The resulting solution was stirred at 80 °C for 15 h. After the reaction was quenched with saturated aqueous NH_4_Cl solution (2 mL), the resulting mixture was extracted with ethyl acetate (10 mL × 3). The combined organic layers were washed with brine (10 mL), dried over Na_2_SO_4_, filtered, and concentrated under reduced pressure. The crude residue was purified by silica-gel column chromatography (hexane/AcOEt = 10/1 as an eluent; *R*_f_ = 0.40) to give the title compound **5b** as a pale-yellow solid (55 mg, 80% yield). The compound **5b** can be separated into enantiomerically-pure (*P*)-**5b** and (*M*)-**5b** by HPLC equipped with a DAICEL CHIRALPAK^®^ IA-3 column (Daicel Corporation, Tokyo, Japan) (4.6 mm × 250 mm) [*t*_R_ = 4.24 min for (*P*)-**5b** and 5.23 min for (*M*)-**5b** (flow rate: 1.0 mL; eluent: Hex/CHCl_3_ = 7/3)]: ^1^H NMR (500 MHz, CDCl_3_) δ 8.03 (d, *J* = 8.6 Hz, 2H), 8.01 (d, *J* = 8.6, 2H), 7.844 (d, *J* = 8.6 Hz, 2H), 7.835 (d, *J* = 7.5 Hz, 2H), 7.78 (d, *J* = 8.6 Hz, 2H), 7.63–7.61 (m, 2H), 7.53 (d, *J* = 8.0 Hz, 2H), 7.50 (d, *J* = 8.6 Hz, 2H), 7.22–7.18 (m, 2H), 6.28–6.25 (m, 2H); ^13^C NMR (126 MHz, CDCl_3_) δ 140.4, 138.7, 134.6, 131.5, 130.9, 130.5, 128.5, 128.3, 127.4, 127.1, 126.9, 126.8, 126.4, 126.1, 124.6, 122.8, 117.2, 110.9, 21.5; HRMS–APCI^+^ (*m/z*) calcd for C_35_H_24_N^+^ ([M+H]^+^) 458.1903, found 458.1904.

#### 3.2.6. 9-(4-Methoxyphenyl)-9*H*-dinaphtho[2,1-*c*:1′,2′-*g*]carbazole (**5c**)

The crude product was obtained by using diphenanthro[3,4-*b*:4′,3′-*d*]thiophene *S*,*S*-dioxide (**1b**) (42 mg, 0.10 mmol), *p*-anisidine (25 mg, 0.20 mmol), 1,4-dioxane (1.5 mL), KN(SiMe_3_)_2_ (0.5 M in toluene, 0.60 mL, 0.30 mmol) at 80 °C for 19 h according to the procedure for **5b**. Purification by silica-gel column chromatography with (hexane/AcOEt = 5/1 as an eluent; *R*_f_ = 0.48) gave the title compound **5b** as a pale-yellow solid (42 mg, 88% yield). The compound **5c** can be separated into enantiomerically-pure (*P*)-**5c** and (*M*)-**5c** by HPLC equipped with a DAICEL CHIRALPAK^®^ IA-3 column (Daicel Corporation, Tokyo, Japan) (4.6 mm × 250 mm) [*t*_R_ = 5.02 min for (*P*)-**5c** and 6.18 min for (*M*)-**5c** (flow rate: 1.0 mL; eluent: Hex/CHCl_3_ = 7/3)]: ^1^H NMR (400 MHz, CDCl_3_) δ 8.02 (d, *J* = 8.2 Hz, 2H), 8.01 (d, *J* = 8.7, 2H), 7.84 (d, *J* = 8.7 Hz, 2H), 7.83 (d, *J* = 7.6 Hz, 2H), 7.73 (d, *J* = 8.2 Hz, 2H), 7.63–7.59 (m, 2H), 7.50 (d, *J* = 8.2 Hz, 2H), 7.23–7.18 (m, 4H), 6.29–6.25 (m, 2H); ^13^C NMR (101 MHz, CDCl_3_) δ 159.8, 140.7, 131.5, 130.5, 129.9, 129.8, 128.4, 127.4, 127.1, 126.94, 126.86, 126.4, 126.1, 124.6, 122.8, 117.1, 115.4, 110.9, 55.9; HRMS–APCI^+^ (*m*/*z*) calcd for C_35_H_24_NO^+^ ([M+H]^+^) 474.1852, found 474.1861.

### 3.3. Computational Studies

The DFT and TD-DFT calculations were performed by using the Gaussian 16 program [43] at the B3LYP/6‒31G(d) level of theory in the gas phase. The starting molecular models for DFT geometry optimizations were built and optimized with MMFF molecular mechanics by using the Spartan ’08 package (Wavefunction, Inc., Irvine, CA, USA). Twelve singlet states were calculated in TD-DFT calculations. The visualization of the molecular orbitals has been performed using GaussView 5 (Gaussian, Inc., Wallingford, CT, USA).

### 3.4. X-ray Crystallography

For X-ray crystallographic analysis, a suitable single crystal was selected under ambient conditions, mounted using a nylon loop filled with paraffin oil, and transferred to the goniometer of a RIGAKU R–AXIS RAPID diffractometer (Rigaku Corporation, Tokyo, Japan) with a graphite-monochromated Cu–Kα irradiation (*λ* = 1.54187 Å). The structure was solved by a direct method (SIR 2008 [44]) and refined by full-matrix least-squares techniques against *F*2 (SHELXL-2014 [45,46]). The intensities were corrected for Lorentz and polarization effects. All non-hydrogen atoms were refined anisotropically. Hydrogen atoms were placed using AFIX instructions.

Crystal Data for (*M*)-**5c**·(CHCl_3_): Formula: C_35_H_23_NO·CHCl_3_ (*M* = 592.91 g/mol): monoclinic, space group *P*2_1_ (No. 4), *a* = 8.25287(15) Å, *b* = 18.2292(3) Å, *c* = 10.12564(18) Å, *β* = 110.7590(8)°, *V* = 1424.44(5) Å^3^, *Z* = 2, *T* = 193(2) K, *μ*(CuKα) = 3.152 mm^−1^, *D*_calc_ = 1.382 g/cm^3^, 22,925 reflections measured (4.670° ≤ θ ≤ 68.238°), 4954 unique (*R*_int_ = 0.0676; *R*_sigma_ = 0.0575), which were used in all calculations. The final *R*_1_ was 0.0536 (*I* > 2σ(*I*)) and w*R*_2_ was 0.1458 (all data). CCDC 2,131,542 contains the supplementary crystallographic data for this paper. The data can be obtained free of charge from The Cambridge Crystallographic Data Centre via www.ccdc.cam.ac.uk/structures

## 4. Conclusions

In summary, we have demonstrated that the thiophene-fused thia[7]helicene can be converted into a series of aza[7]helicenes and [7]helicene-like compounds via aromatic metamorphosis without a transition metal catalyst. Especially, the 3,3′-dianion of 4,4′-biphenanthrene formed by desulfurative dilithiation were found to react with heteroatom electrophiles to give the corresponding ring-closing product. The dianion can also be prepared from 3,3′-dibromo-4,4′-biphenanthrene [37]. However, the yield of the dibromide from 4,4′-biphenanthrene-3,3′-diol is relatively low (16% for two steps). Therefore, the present approach would be a convenient alternative for the preparation of the 3,3′-dianion. Further efforts are focused on applying this methodology to other classes of thiophene-fused thiahelicenes, electrophiles, and nucleophiles to develop novel helicenes with unique properties.

## Figures and Tables

**Table 1:** Photophysical properties of [7]helicene-like compounds and aza[7]helicenes.

	λ_abs_ (nm) *^a^*	λ_em_ (nm) *^b^*	ϕ *^c^*
**1a**	401	417, 435 (sh) (265)	<1
**1b**	395, 415 (sh)	458 (300)	4
**2b**	389, 411 (sh)	434 (sh), 454 (325)	11
**4b**	406	422, 441 (sh) (294)	31
**5b**	401	421, 444 (335)	17
**5c**	401	422, 445 (335)	16
**2a** ***^d^***	412	450 (320)	23
**4a** ***^e^***	408	421, 441 (sh) (340)	40
**5a** ***^f^***	400	418, 442 (330)	17

*^a^* The longest absorption maximum in CH_2_Cl_2_. sh: shoulder. *^b^* Emission maximum in CH_2_Cl_2_. Excitation wavelength in parenthesis. *^c^* Absolute quantum yield in CH_2_Cl_2_. *^d^* Reference [35]. *^e^* Reference [36]. *^f^* Reference [33].

## Data Availability

The data presented in this study are available in Supplementary Material.

## Supplementary Materials

The following supporting information can be downloaded. Figures S1–S8: ^1^H and ^13^C NMR spectra for **2b**, **4b**, **5b**, and **5c**; Table S1: Crystallographic data for (*M*)-**5c**; Tables S2–S10: Coordinates and absolute energy of the optimized structures for (*P*)-**1a**–**1b**, (*P*)-**2a**–**2b**, (*P*)-**4a**–**4b**, and (*P*)-**5a**–**5c**; Figures S9–S13: Molecular orbitals for (*P*)-**1a**–**1b**, (*P*)-**2a**–**2b**, (*P*)-**4a**–**4b**, and (*P*)-**5a**–**5c**; Table S11: The selected absorption of **1**, **2**, **4** and **5** calculated by TD–DFT method; File S1: CIF file for (*M*)-**5c**.

## Sample Availability

Samples of the compounds are available from the authors.

## References

[1] Shen Y., Chen C.F. (2012). Helicenes: Synthesis and Applications. Chem. Rev..

[2] Gingras M. (2013). One hundred years of helicene chemistry. Part 3: Applications and properties of carbohelicenes. Chem. Soc. Rev..

[3] Gingras M., Félix G., Peresutti R. (2013). One hundred years of helicene chemistry. Part 2: Stereoselective syntheses and chiral separations of carbohelicenes. Chem. Soc. Rev..

[4] Verbiest T., Elshocht Sven V., Kauranen M., Hellemans L., Snauwaert J., Nuckolls C., Katz Thomas J., Persoons A. (1998). Strong Enhancement of Nonlinear Optical Properties Through Supramolecular Chirality. Science.

[5] Hatakeyama T., Hashimoto S., Oba T., Nakamura M. (2012). Azaboradibenzo[6]helicene: Carrier inversion induced by helical homochirality. J. Am. Chem. Soc..

[6] Zhang L., Song I., Ahn J., Han M., Linares M., Surin M., Zhang H.J., Oh J.H., Lin J. (2021). π-Extended perylene diimide double-heterohelicenes as ambipolar organic semiconductors for broadband circularly polarized light detection. Nat. Commun..

[7] Hua W., Liu Z., Duan L., Dong G., Qiu Y., Zhang B., Cui D., Tao X., Cheng N., Liu Y. (2015). Deep-blue electroluminescence from nondoped and doped organic light-emitting diodes (OLEDs) based on a new monoaza[6]helicene. RSC Adv..

[8] Aillard P., Voituriez A., Dova D., Cauteruccio S., Licandro E., Marinetti A. (2014). Phosphathiahelicenes: Synthesis and uses in enantioselective gold catalysis. Chem. Eur. J..

[9] Oestreich M., Gross B.M. (2021). The Trityl Cation Embedded into a [7]Helicene-Like Backbone: Preparation and Application as a Lewis Acid Catalyst. Synthesis.

[10] Zhao W.L., Li M., Lu H.Y., Chen C.F. (2019). Advances in helicene derivatives with circularly polarized luminescence. Chem. Commun..

[11] Flammang-Barbieux M., Nasielski J., Martin R.H. (1967). Synthesis of heptahelicene (1) benzo [c] phenanthro [4, 3-g] phenanthrene. Tetrahedron Lett..

[12] Willmore N.D., Liu L.B., Katz T.J. (1992). A Diels-Alder Route to [5]-Helicenes and [6]-Helicenes. Angew. Chem. Int. Ed..

[13] Ichikawa J., Yokota M., Kudo T., Umezaki S. (2008). Efficient helicene synthesis: Friedel-Crafts-type cyclization of 1,1-difluoro-1-alkenes. Angew. Chem. Int. Ed..

[14] Stará I.G., Starý I., Kollárovič A., Teplý F., Šaman D., Tichý M. (1998). A novel strategy for the synthesis of molecules with helical chirality. Intramolecular [2+2+2] cycloisomerization of triynes under cobalt catalysis. J. Org. Chem..

[15] Tanaka K., Kamisawa A., Suda T., Noguchi K., Hirano M. (2007). Rh-catalyzed synthesis of helically chiral and ladder-type molecules via [2+2+2] and formal [2+1+2+1] cycloadditions involving C-C triple bond cleavage. J. Am. Chem. Soc..

[16] Tanaka K., Fukawa N., Suda T., Noguchi K. (2009). One-step construction of five successive rings by rhodium-catalyzed intermolecular double [2+2+2] cycloaddition: Enantioenriched [9]helicene-like molecules. Angew. Chem. Int. Ed..

[17] Sawada Y., Furumi S., Takai A., Takeuchi M., Noguchi K., Tanaka K. (2012). Rhodium-Catalyzed Enantioselective Synthesis, Crystal Structures, and Photophysical Properties of Helically Chiral 1,1′-Bitriphenylenes. J. Am. Chem. Soc..

[18] Storch J., Bernard M., Sýkora J., Karban J., Čermák J. (2013). Intramolecular Cascade Hydroarylation/Cycloisomerization Strategy for the Synthesis of Polycyclic Aromatic and Heteroaromatic Systems. Eur. J. Org. Chem..

[19] Shimizu M., Nagao I., Tomioka Y., Hiyama T. (2008). Palladium-catalyzed annulation of vic-bis(pinacolatoboryl)alkenes and -phenanthrenes with 2,2′-dibromobiaryls: Facile synthesis of functionalized phenanthrenes and dibenzo[*g*,*p*]chrysenes. Angew. Chem. Int. Ed..

[20] Collins S.K., Grandbois A., Vachon M.P., Côté J. (2006). Preparation of helicenes through olefin metathesis. Angew. Chem. Int. Ed..

[21] Grandbois A., Collins S.K. (2008). Enantioselective Synthesis of [7]Helicene: Dramatic Effects of Olefin Additives and Aromatic Solvents in Asymmetric Olefin Metathesis. Chem. Eur. J..

[22] Bhanuchandra M., Murakami K., Vasu D., Yorimitsu H., Osuka A. (2015). Transition-Metal-Free Synthesis of Carbazoles and Indoles by an S_N_Ar-Based “Aromatic Metamorphosis” of Thiaarenes. Angew. Chem. Int. Ed..

[23] Yorimitsu H., Vasu D., Bhanuchandra M., Murakami K., Osuka A. (2016). Aromatic Metamorphosis of Dibenzothiophenes. Synlett.

[24] Bhanuchandra M., Yorimitsu H., Osuka A. (2016). Synthesis of Spirocyclic Diarylfluorenes by One-Pot Twofold S_N_Ar Reactions of Diaryl Sulfones with Diarylmethanes. Org. Lett..

[25] Kaga A., Nogi K., Yorimitsu H. (2019). Synthesis of N-Alkyl and N-H-Carbazoles through S_N_Ar-Based Aminations of Dibenzothiophene Dioxides. Chem. Eur. J..

[26] Kaga A., Iida H., Tsuchiya S., Saito H., Nakano K., Yorimitsu H. (2021). Aromatic Metamorphosis of Thiophenes by Means of Desulfurative Dilithiation. Chem. Eur. J..

[27] Nagata Y., Kato S., Miyake Y., Shinokubo H. (2017). Synthesis of Tetraaza[8]circulenes from Tetrathia[8]circulenes through an SNAr-Based Process. Org. Lett..

[28] Matsuo Y., Tanaka T., Osuka A. (2020). Diazadimethano[8]circulene: Synthesis, Structure, Properties, and Isolation of Stable Radical Cation. Chem. Lett..

[29] Takase K., Noguchi K., Nakano K. (2019). Synthesis of Pyrrole-Containing Chiral Spiro Molecules and Their Optical and Chiroptical Properties. Bull. Chem. Soc. Jpn..

[30] Yanagi T., Tanaka T., Yorimitsu H. (2021). Asymmetric systematic synthesis, structures, and (chir)optical properties of a series of dihetero[8]helicenes. Chem. Sci..

[31] Dore A., Fabbri D., Gladiali S., Valle G. (1995). New axially chiral sulfur compounds: Synthesis and conformational stability of enantiopure 4,4′-biphenanthrene-3,3′-dithiol and related atropisomeric derivatives. Tetrahedron Asymmetry.

[32] Cossu S., De Lucchi O., Fabbri D., Valle G., Painter G.F., Smith R.A.J. (1997). Synthesis of Structurally Modified Atropisomeric Biaryl Dithiols. Observations on the Newman-Kwart Rearrangement. Tetrahedron.

[33] Nakano K., Hidehira Y., Takahashi K., Hiyama T., Nozaki K. (2005). Stereospecific synthesis of hetero[7]helicenes by Pd-catalyzed double *N*-arylation and intramolecular *O*-arylation. Angew. Chem. Int. Ed..

[34] Nakano K., Oyama H., Nishimura Y., Nakasako S., Nozaki K. (2012). λ^5^-Phospha[7]helicenes: Synthesis, Properties, and Columnar Aggregation with One-Way Chirality. Angew. Chem. Int. Ed..

[35] Oyama H., Nakano K., Harada T., Kuroda R., Naito M., Nobusawa K., Nozaki K. (2013). Facile Synthetic Route to Highly Luminescent Sila[7]helicene. Org. Lett..

[36] Oyama H., Akiyama M., Nakano K., Naito M., Nobusawa K., Nozaki K. (2016). Synthesis and Properties of [7]Helicene-like Compounds Fused with a Fluorene Unit. Org. Lett..

[37] Terada N., Uematsu K., Higuchi R., Tokimaru Y., Sato Y., Nakano K., Nozaki K. (2021). Synthesis and Properties of Spiro-double Sila[7]helicene: The LUMO Spiro-conjugation. Chem. Eur. J..

[38] Fukui N., Osuka A. (2018). Singly and Doubly Sulfone-Inserted Porphyrin Arch-Tape Dimers. Bull. Chem. Soc. Jpn..

[39] Simmons H.E., Fukunaga T. (1967). Spiroconjugation. J. Am. Chem. Soc..

[40] Schweig A., Weulner U., Hellwinkel D., Krapp W. (1973). Spiroconjugation. Angew. Chem. Int. Ed..

[41] Dürr H., Gleiter R. (1978). Spiroconjugation. Angew. Chem. Int. Ed..

[42] Sanchez-Carnerero E.M., Agarrabeitia A.R., Moreno F., Maroto B.L., Muller G., Ortiz M.J., de la Moya S. (2015). Circularly Polarized Luminescence from Simple Organic Molecules. Chem. Eur. J..

[43] Frisch M.J., Trucks G.W., Schlegel H.B., Scuseria G.E., Robb M.A., Cheeseman J.R., Scalmani G., Barone V., Petersson G.A., Nakatsuji H. (2016). Gaussian 16 Rev. C.01.

[44] Burla M.C., Caliandro R., Camalli M., Carrozzini B., Cascarano G.L., De Caro L., Giacovazzo C., Polidori G., Siliqi D., Spagna R. (2007). IL MILIONE: A suite of computer programs for crystal structure solution of proteins. J. Appl. Crystallogr..

[45] Sheldrick G.M. (2008). A short history of SHELX. Acta Crystallogr A.

[46] Sheldrick G.M. (2015). Crystal structure refinement with SHELXL. Acta Crystallogr. C Struct. Chem..

