# Supplementary material for: Transformation of Thia[7]helicene to Aza[7]helicenes and [7]Helicene-like Compounds via Aromatic Metamorphosis
Source: Molecules. 2022 Jan 18;27(3):606. doi: 10.3390/molecules27030606 (PMC8839769; doi:10.3390/molecules27030606)
Supplement: Supplementary file 1 [file molecules-27-00606-s001.zip › molecules-1563227-supplementary.pdf]

# Supporting Information

## Transformation of Thia[7]helicene to Aza[7]helicenes and [7]Helicene-like Compounds via Aromatic Metamorphosis

Keisuke Uematsu [1], Chikara Hayasaka [1], Ko Takase [1], Keiichi Noguchi [2], Koji Nakano [1,*]

[1]*Department of Applied Chemistry, Graduate School of Engineering, Tokyo University of Agriculture and Technology, 2-24-16 Naka-cho, Koganei, Tokyo 184-8588, Japan*
[2]*Instrumentation Analysis Center, Tokyo University of Agriculture and Technology, 2-24-16 Naka-cho, Koganei, Tokyo 184-8588, Japan*

e-mail: k_nakano@cc.tuat.ac.jp

## Table of Contents

$^{1}H$ and $^{13}C$ NMR Spectra of **2b**, **4b**, **5b**, and **5c** S3−S6

X-ray Analysis S7

DFT and TD-DFT Calculation Results S8−S21

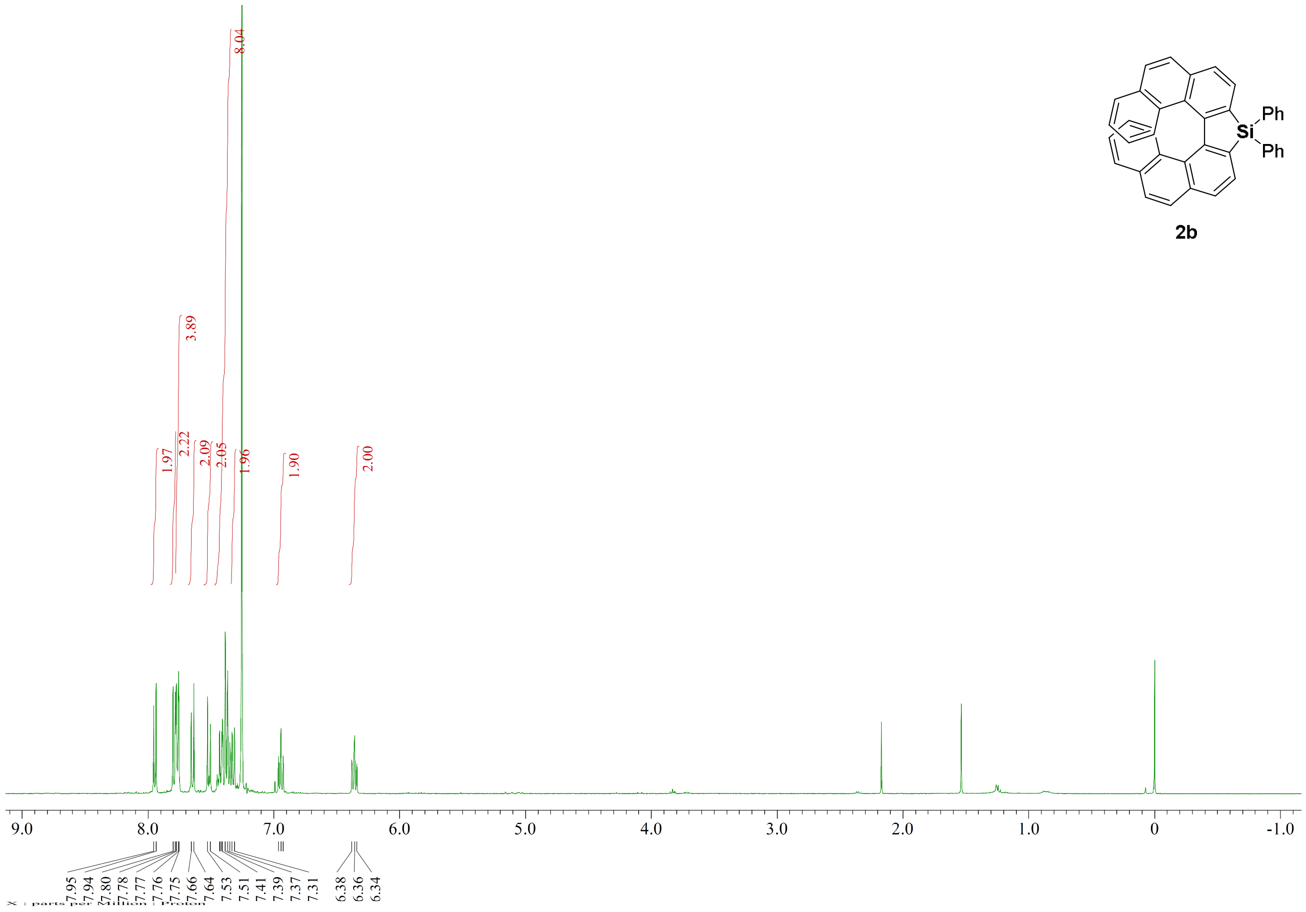


**Figure S1.** $^{1}H$ NMR spectrum of **2b** (400 MHz, $CDCl_3$).

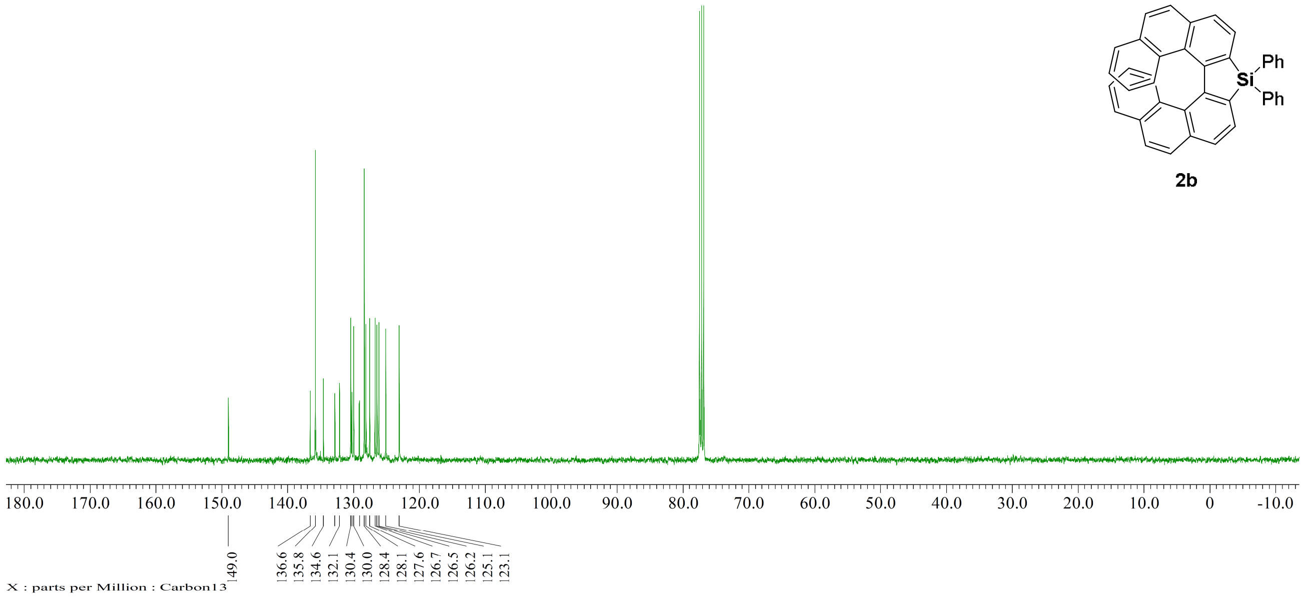


**Figure S2.** $^{13}C$ NMR spectrum of **2b** (101 MHz, $CDCl_3$).

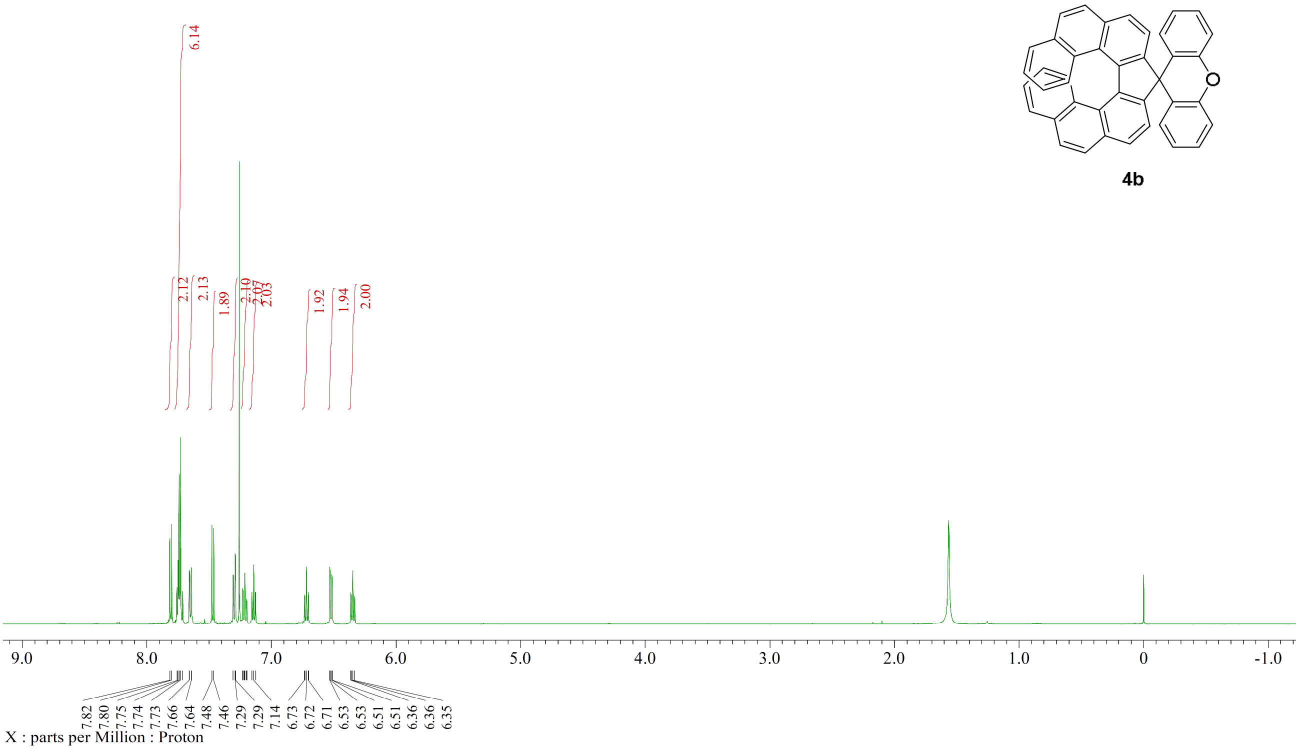


**Figure S3.** $^{1}$H NMR spectrum of **4b** (500 MHz, $CDCl_3$).

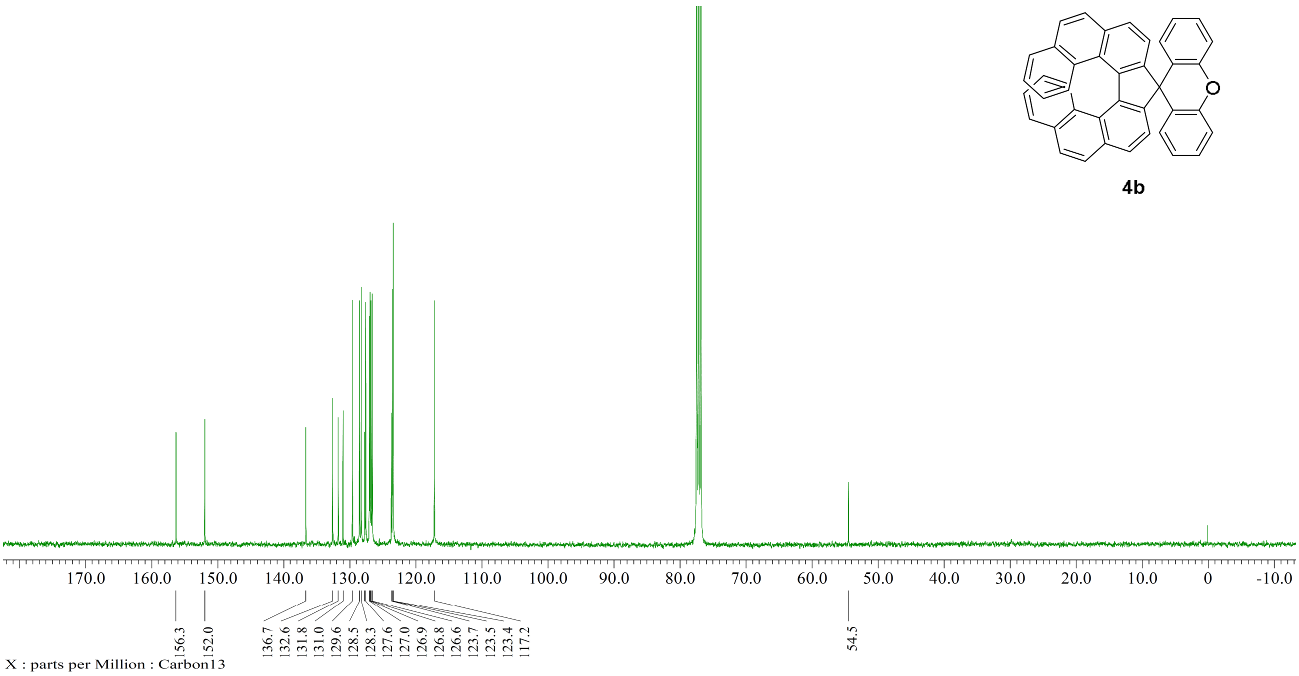


**Figure S4.** $^{13}$C NMR spectrum of **4b** (101 MHz, $CDCl_3$).

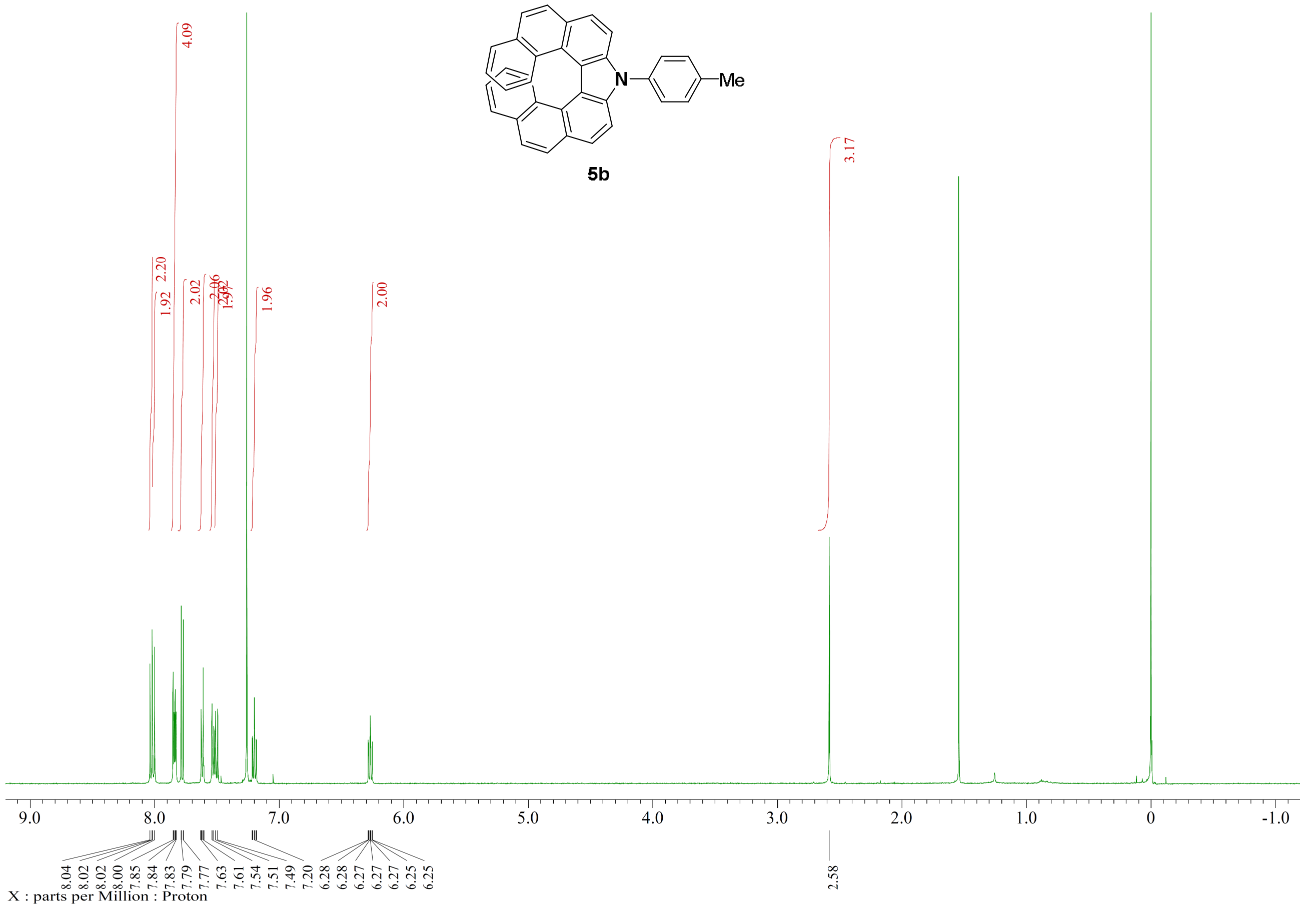


**Figure S5.** $^{1}$H NMR spectrum of **5b** (500 MHz, $CDCl_3$).

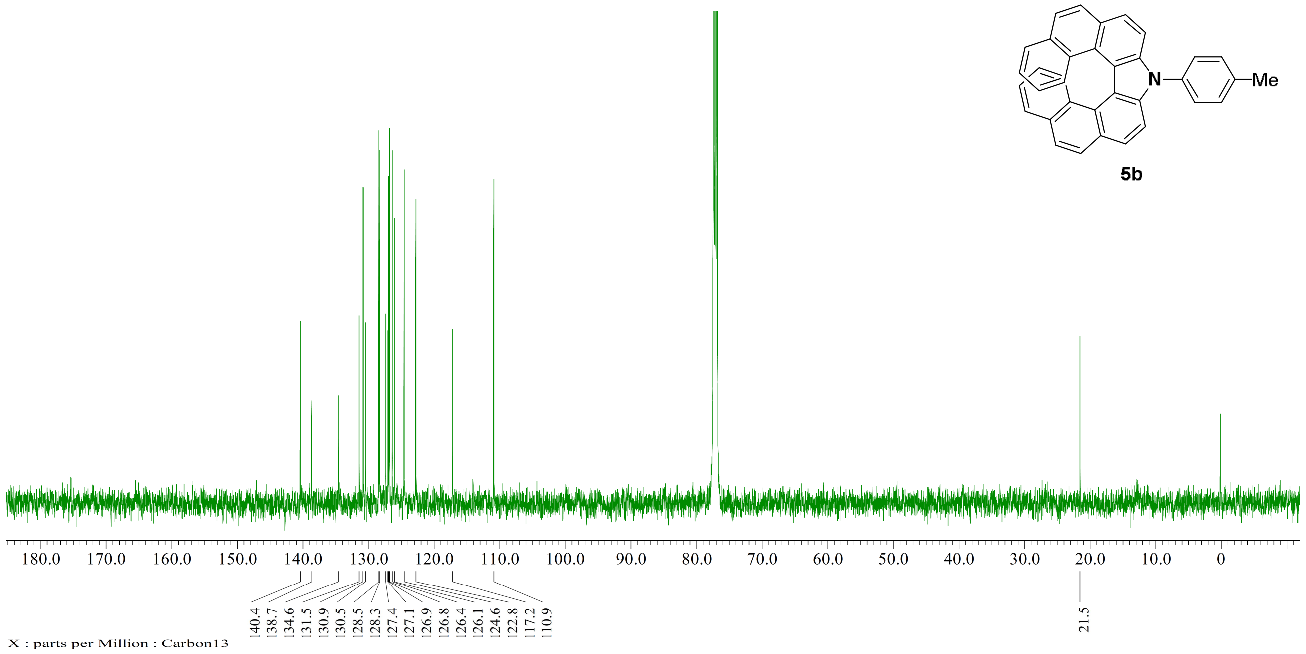


**Figure S6.** $^{13}$C NMR spectrum of **5b** (126 MHz, $CDCl_3$).

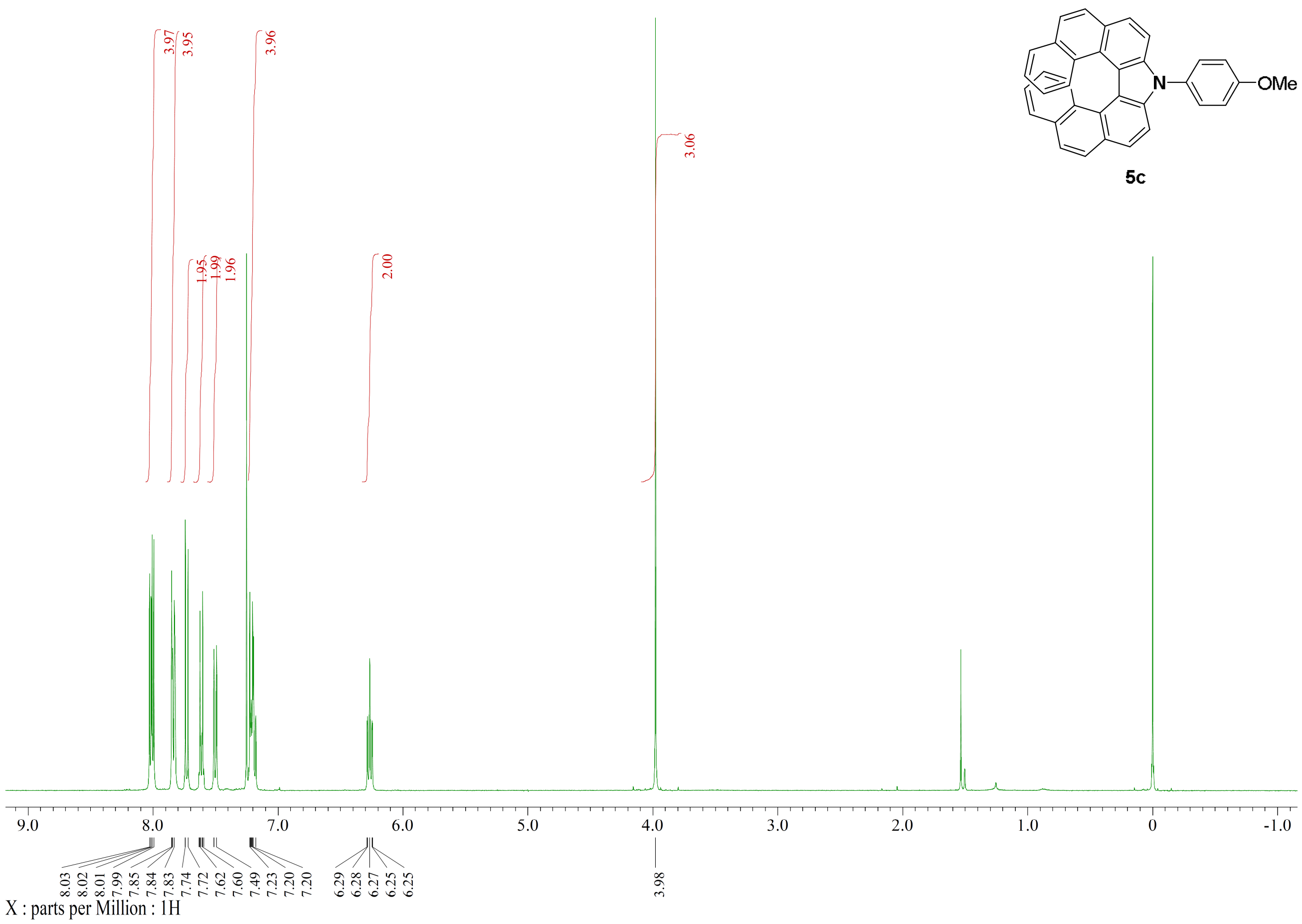

**Figure S7.** $^{1}$H NMR spectrum of **5c** (400 MHz, $CDCl_3$).

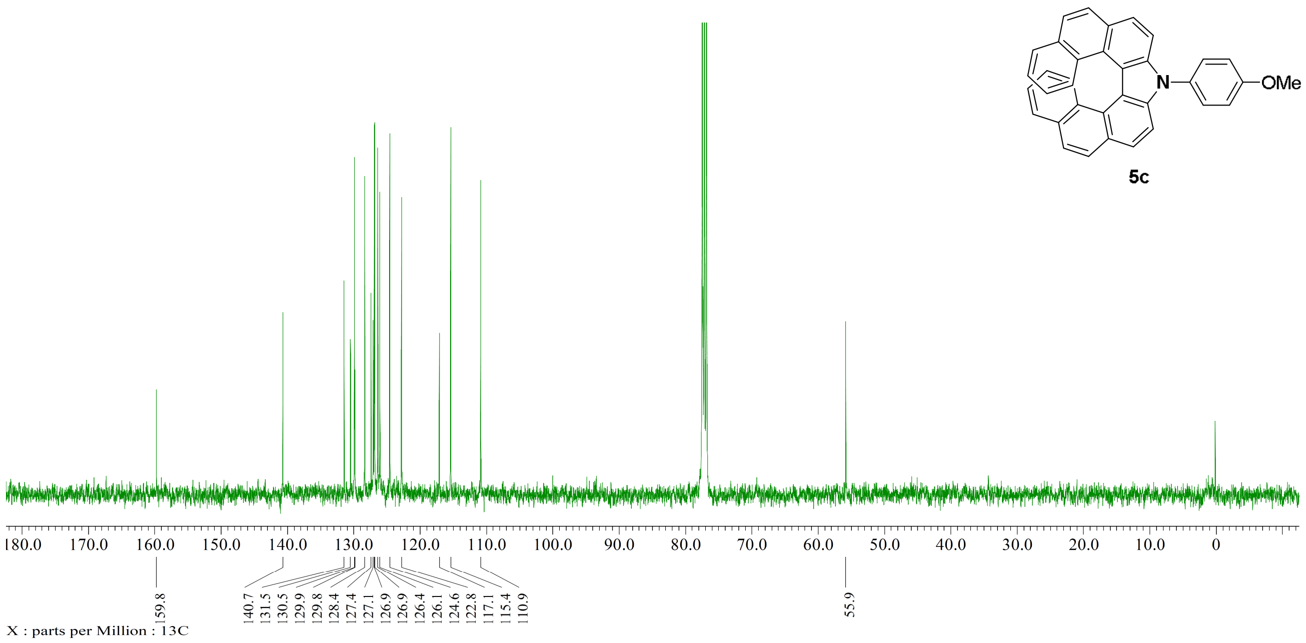

**Figure S8.** $^{13}$C NMR spectrum of **5c** (101 MHz, $CDCl_3$).

## X-ray Crystallography

**Table S1.** Crystallographic Data and Structure Refinement Details for (*M*)-**5c**

| | | |
|---|---|---|
| Formula | $(C_{35}H_{23}NO)\bullet(CHCl_3)$ | |
| Formula weight | 592.91 | |
| Temperature | 193(2) K | |
| Wavelength | 1.54187 Å | |
| Crystal system | monoclinic | |
| Space group | $P2_1$ | |
| Unit cell dimensions | $a$ = 8.25287(15) Å | $\alpha$ = 90° |
| | $b$ = 18.2292(3) Å | $\beta$ =110.7590(8)° |
| | $c$ = 10.12564(18) Å | $\gamma$ = 90° |
| Volume | 1424.44(5) $Å^3$ | |
| $Z$ | 2 | |
| Density (calculated) | 1.382 $g/cm^3$ | |
| Absorption coefficient | 3.152 $mm^{-1}$ | |
| $F$(000) | 612 | |
| Crystal size | $0.60 \times 0.40 \times 0.10$ $mm^3$ | |
| Theta range for data collection | 4.670 to 68.238° | |
| Index ranges | –9<=h<=9, –21<=k<=21, –12<=l<=12 | |
| Reflections collected | 22925 | |
| Independent reflections | 4954 [$R_{int}$ = 0.0676] | |
| Completeness to theta | 99.7% | |
| Max. and min. transmission | 0.730 and 0.234 | |
| Refinement method | Full-matrix least-squares on $F^2$ | |
| Data / restraints / parameters | 4954 / 1 / 370 | |
| Goodness-of-fit on $F^2$ | 1.045 | |
| Flack parameter | 0.003(10) | |
| Final $R$ indices [$I$>2sigma($I$)] | $R_1$ = 0.0536, w$R_2$ = 0.1365 | |
| $R$ indices (all data) | $R_1$ = 0.0584, w$R_2$ = 0.1458 | |
| Largest diff. peak and hole | 0.429 and –0.456 e/$Å^3$ | |

## Computational Studies

**Table S2**. Coordinates (Å) and Absolute Energy of the Optimized Structure for (*P*)-**1a**

| atom | x | y | z | atom | x | y | z |
|---|---|---|---|---|---|---|---|
| H | -2.83377909 | 4.07890576 | -0.87405005 | C | 2.53018561 | 3.0557608 | 0.6745414 |
| C | -2.53040383 | 3.05558379 | -0.67454021 | H | 4.40743515 | 2.17867378 | 1.20924985 |
| H | -4.4075943 | 2.17836568 | -1.20924112 | C | 3.40014252 | 2.00575605 | 0.83959519 |
| C | -3.40028821 | 2.00551833 | -0.83959022 | C | 0.73445206 | 1.45361579 | 0.06384916 |
| C | -0.73455647 | 1.45356441 | -0.06385298 | C | 3.0334022 | 0.69041714 | 0.45314904 |
| C | -3.03345484 | 0.69020521 | -0.45314453 | C | 1.2135877 | 2.76546069 | 0.27858557 |
| C | -1.21378448 | 2.7653758 | -0.27858797 | C | 1.73038619 | 0.41442702 | -0.0640666 |
| C | -1.73041749 | 0.41430624 | 0.06406584 | C | 4.00408654 | -0.36021256 | 0.55225428 |
| C | -4.00406639 | -0.36049228 | -0.55224464 | H | -0.25261064 | -0.31083594 | -1.83609794 |
| H | 0.25264152 | -0.3108188 | 1.83608303 | C | 3.74908068 | -1.60772885 | 0.07601551 |
| C | -3.74897147 | -1.60799046 | -0.07600598 | H | 4.95859022 | -0.12957067 | 1.01911794 |
| H | -4.95858823 | -0.12991727 | -1.0191042 | H | 4.48599667 | -2.40160413 | 0.16937377 |
| H | -4.48583251 | -2.4019172 | -0.16936042 | C | 2.54132434 | -1.8718499 | -0.64415328 |
| C | -2.54119309 | -1.87202709 | 0.64415688 | C | 2.36450104 | -3.10757218 | -1.31007664 |
| C | -2.36427977 | -3.10773689 | 1.31007947 | C | 1.54329964 | -0.85298719 | -0.75907483 |
| C | -1.54323889 | -0.8530948 | 0.75907292 | C | 1.27540082 | -3.32771889 | -2.12791467 |
| C | -1.27515916 | -3.32780765 | 2.12791087 | H | 3.1262506 | -3.8742922 | -1.18864677 |
| H | -3.12597652 | -3.87451007 | 1.18865411 | H | 1.15808264 | -4.27701168 | -2.64350322 |
| H | -1.15777168 | -4.27709227 | 2.64349871 | C | 0.33646666 | -2.29675325 | -2.3183772 |
| C | -0.33629521 | -2.29677687 | 2.31836712 | H | -0.49746389 | -2.4407803 | -2.9998012 |
| H | 0.49764986 | -2.44074605 | 2.99978558 | C | 0.46900694 | -1.09462273 | -1.64954966 |
| C | -0.4689233 | -1.09465563 | 1.64954033 | S | -0.00014129 | 3.99839146 | -0.00000221 |
| H | 2.83348871 | 4.07910397 | 0.87405221 | | | | |
| absolute energy *E* (B3LYP): -1474.85038702 au | | | | | | | |

[a]Calculated by DFT method [B3LYP/6-31G(d)]

**Table S3**. Coordinates (Å) and Absolute Energy of the Optimized Structure for (*P*)-**1b**

| atom | x | y | z | atom | x | y | z |
|---|---|---|---|---|---|---|---|
| H | -1.0999786 | -2.78391326 | 3.70381277 | H | 1.46889361 | 4.34694669 | 1.77642759 |
| C | -0.86349438 | -2.49740806 | 2.68410032 | C | 1.03833485 | 3.36147307 | 1.62170244 |
| H | -1.46889361 | -4.34694669 | 1.77642759 | C | 0.10980265 | 0.74479673 | 1.10625799 |
| C | -1.03833485 | -3.36147307 | 1.62170244 | C | 0.59633885 | 3.00768786 | 0.32678981 |
| C | -0.10980265 | -0.74479673 | 1.10625799 | C | 0.4110009 | 1.21294313 | 2.39035046 |
| C | -0.59633885 | -3.00768786 | 0.32678981 | C | 0.00000000 | 1.72725751 | 0.06821291 |
| C | -0.4110009 | -1.21294313 | 2.39035046 | C | 0.73345643 | 3.95819504 | -0.74120593 |
| C | 0.00000000 | -1.72725751 | 0.06821291 | H | -1.89153404 | -0.16348922 | -0.61255936 |
| C | -0.73345643 | -3.95819504 | -0.74120593 | C | 0.22855614 | 3.70996573 | -1.97699505 |
| H | 1.89153404 | 0.16348922 | -0.61255936 | H | 1.25460905 | 4.88851005 | -0.53104203 |
| C | -0.22855614 | -3.70996573 | -1.97699505 | H | 0.35163116 | 4.43030537 | -2.78185052 |
| H | -1.25460905 | -4.88851005 | -0.53104203 | C | -0.56025158 | 2.53810842 | -2.22099943 |
| H | -0.35163116 | -4.43030537 | -2.78185052 | C | -1.24702739 | 2.38601097 | -3.44695184 |
| C | 0.56025158 | -2.53810842 | -2.22099943 | C | -0.72031243 | 1.56124549 | -1.1906256 |
| C | 1.24702739 | -2.38601097 | -3.44695184 | C | -2.12517965 | 1.33922798 | -3.64626394 |
| C | 0.72031243 | -1.56124549 | -1.1906256 | H | -1.09390293 | 3.13179694 | -4.22329786 |
| C | 2.12517965 | -1.33922798 | -3.64626394 | H | -2.65690022 | 1.24038288 | -4.58851171 |
| H | 1.09390293 | -3.13179694 | -4.22329786 | C | -2.35530773 | 0.42408329 | -2.60381032 |

| | | | | | | | |
|---|---|---|---|---|---|---|---|
| H | 2.65690022 | -1.24038288 | -4.58851171 | H | -3.08303554 | -0.37241656 | -2.7308888 |
| C | 2.35530773 | -0.42408329 | -2.60381032 | C | -1.6693337 | 0.53538331 | -1.40777959 |
| H | 3.08303554 | 0.37241656 | -2.7308888 | S | 0.00000000 | 0.00000000 | 3.63829461 |
| C | 1.6693337 | -0.53538331 | -1.40777959 | O | -1.18101124 | 0.45669748 | 4.38810747 |
| H | 1.0999786 | 2.78391326 | 3.70381277 | O | 1.18101124 | -0.45669748 | 4.38810747 |
| C | 0.86349438 | 2.49740806 | 2.68410032 | | | | |
| absolute energy *E* (B3LYP): -1625.20986910 au | | | | | | | |

[a]Calculated by DFT method [B3LYP/6-31G(d)]

**Table S4**. Coordinates (Å) and Absolute Energy of the Optimized Structure for (*P*)-**2a**

| atom | x | y | z | atom | x | y | z |
|---|---|---|---|---|---|---|---|
| H | 1.38223688 | 2.87788072 | 3.48256236 | C | -0.6646502 | -2.93651246 | 0.15404309 |
| C | 1.06159857 | 2.52182727 | 2.5057669 | C | -0.53866915 | -1.22664477 | 2.36185319 |
| H | 1.70159306 | 4.2966335 | 1.4690102 | C | -0.00000000 | -1.67419265 | 0.01279868 |
| C | 1.21246426 | 3.32813514 | 1.39516991 | C | -0.77002819 | -3.8209215 | -0.97278783 |
| C | 0.14262288 | 0.74172818 | 1.09436024 | H | 2.01732661 | 0.15804509 | -0.44325894 |
| C | 0.6646502 | 2.93651246 | 0.15404309 | C | -0.17574881 | -3.52992492 | -2.15848952 |
| C | 0.53866915 | 1.22664477 | 2.36185319 | H | -1.34475995 | -4.73579984 | -0.8500423 |
| C | -0.00000000 | 1.67419265 | 0.01279868 | H | -0.27659519 | -4.19795015 | -3.01052958 |
| C | 0.77002819 | 3.8209215 | -0.97278783 | C | 0.6762269 | -2.38355595 | -2.283425 |
| H | -2.01732661 | -0.15804509 | -0.44325894 | C | 1.446368 | -2.19588953 | -3.45419604 |
| C | 0.17574881 | 3.52992492 | -2.15848952 | C | 0.80965607 | -1.47455694 | -1.1888904 |
| H | 1.34475995 | 4.73579984 | -0.8500423 | C | 2.37946549 | -1.18192913 | -3.53971568 |
| H | 0.27659519 | 4.19795015 | -3.01052958 | H | 1.30981983 | -2.88960495 | -4.28069925 |
| C | -0.6762269 | 2.38355595 | -2.283425 | H | 2.97262962 | -1.05489761 | -4.44129505 |
| C | -1.446368 | 2.19588953 | -3.45419604 | C | 2.58164883 | -0.33898698 | -2.43257253 |
| C | -0.80965607 | 1.47455694 | -1.1888904 | H | 3.34878639 | 0.42977088 | -2.46767703 |
| C | -2.37946549 | 1.18192913 | -3.53971568 | C | 1.81728641 | -0.48606033 | -1.28876275 |
| H | -1.30981983 | 2.88960495 | -4.28069925 | Si | -0.00000000 | -0.00000000 | 3.67769978 |
| H | -2.97262962 | 1.05489761 | -4.44129505 | C | 1.37076525 | -0.7195518 | 4.76444345 |
| C | -2.58164883 | 0.33898698 | -2.43257253 | H | 0.98819563 | -1.52143655 | 5.40768363 |
| H | -3.34878639 | -0.42977088 | -2.46767703 | H | 1.7999455 | 0.04956504 | 5.41829485 |
| C | -1.81728641 | 0.48606033 | -1.28876275 | H | 2.18005407 | -1.13456151 | 4.15447842 |
| H | -1.38223688 | -2.87788072 | 3.48256236 | C | -1.37076525 | 0.7195518 | 4.76444345 |
| C | -1.06159857 | -2.52182727 | 2.5057669 | H | -2.18005407 | 1.13456151 | 4.15447842 |
| H | -1.70159306 | -4.2966335 | 1.4690102 | H | -0.98819563 | 1.52143655 | 5.40768363 |
| C | -1.21246426 | -3.32813514 | 1.39516991 | H | -1.7999455 | -0.04956504 | 5.41829485 |
| C | -0.14262288 | -0.74172818 | 1.09436024 | | | | |
| absolute energy *E* (B3LYP): -1446.00289057 au | | | | | | | |

[a]Calculated by DFT method [B3LYP/6-31G(d)]

**Table S5**. Coordinates (Å) and Absolute Energy of the Optimized Structure for (*P*)-**2b**

| atom | x | y | z | atom | x | y | z |
|---|---|---|---|---|---|---|---|
| H | -2.16432062 | 1.88606563 | 2.56913479 | H | 4.33539232 | -3.55834213 | -2.23683624 |
| C | -1.18613085 | 1.72510314 | 2.12205642 | C | 3.60887251 | -2.41442085 | -0.53623211 |
| H | -0.149066 | 2.98041894 | 3.53000429 | C | 4.78119418 | -2.61176432 | 0.22904487 |
| C | -0.07468909 | 2.36051884 | 2.63964571 | C | 2.51368964 | -1.6799414 | 0.01415716 |
| C | 0.22819017 | 0.58807381 | 0.47366258 | C | 4.86731391 | -2.16146337 | 1.53136236 |
| C | 1.16785743 | 2.27237853 | 1.97452713 | H | 5.60829991 | -3.15591646 | -0.22124081 |

| | | | | | | | |
|---|---|---|---|---|---|---|---|
| C | -1.03990451 | 0.83359595 | 1.04735094 | H | 5.77001982 | -2.32901636 | 2.11260693 |
| C | 1.31047784 | 1.47474537 | 0.79164272 | C | 3.75949887 | -1.51795288 | 2.11090642 |
| C | 2.29561603 | 2.99827176 | 2.48861182 | H | 3.79521669 | -1.20547106 | 3.15097057 |
| H | 1.7677606 | 0.81788882 | -1.85293183 | C | 2.61403146 | -1.28746377 | 1.3699335 |
| C | 3.48269375 | 3.02029716 | 1.82963573 | Si | -2.3579801 | -0.00000732 | 0.00000127 |
| H | 2.17241191 | 3.53190812 | 3.42794952 | C | -3.44055515 | -1.27940225 | 0.86889502 |
| H | 4.33532144 | 3.55847675 | 2.23678879 | C | -4.99763993 | -3.16116425 | 2.28840583 |
| C | 3.60883395 | 2.41447104 | 0.53622726 | C | -3.02863745 | -2.61982299 | 0.99232215 |
| C | 4.78115915 | 2.61179447 | -0.22904936 | C | -4.6558441 | -0.90952363 | 1.47653769 |
| C | 2.51366854 | 1.67994803 | -0.01413711 | C | -5.42602142 | -1.83702744 | 2.17993518 |
| C | 4.86730036 | 2.16142154 | -1.53134103 | C | -3.79706893 | -3.55111564 | 1.69226874 |
| H | 5.60825176 | 3.15598364 | 0.22121563 | H | -2.09747568 | -2.94173279 | 0.53344775 |
| H | 5.77000997 | 2.32895686 | -2.11258489 | H | -5.01289424 | 0.11437792 | 1.39290904 |
| C | 3.75950596 | 1.51785168 | -2.11085939 | H | -6.36098285 | -1.52686908 | 2.6399378 |
| H | 3.79524338 | 1.20530576 | -3.15090361 | H | -3.45916456 | -4.58138909 | 1.77083529 |
| C | 2.61403443 | 1.28738408 | -1.36988616 | H | -5.59732562 | -3.88586544 | 2.83331667 |
| H | -2.16427804 | -1.88604531 | -2.56915316 | C | -3.44056498 | 1.27937436 | -0.86889963 |
| C | -1.18609401 | -1.72508083 | -2.12206296 | C | -4.99766304 | 3.16111747 | -2.28842082 |
| H | -0.14900024 | -2.98033931 | -3.53004006 | C | -3.02866303 | 2.61980025 | -0.9923234 |
| C | -0.07463876 | -2.36046225 | -2.63966414 | C | -4.65584507 | 0.90948083 | -1.4765509 |
| C | 0.22820097 | -0.58806415 | -0.47363759 | C | -5.42602889 | 1.8369753 | -2.17995357 |
| C | 1.16790375 | -2.27231408 | -1.97453994 | C | -3.7971011 | 3.55108359 | -1.69227513 |
| C | -1.03988612 | -0.83358948 | -1.0473423 | H | -2.09750849 | 2.94172143 | -0.53344229 |
| C | 1.31050449 | -1.47471564 | -0.79162906 | H | -5.01288307 | -0.11442519 | -1.3929251 |
| C | 2.2956777 | -2.99816717 | -2.48864666 | H | -6.36098322 | 1.52680548 | -2.63996289 |
| H | 1.7677416 | -0.81802306 | 1.85300176 | H | -3.45920896 | 4.58136126 | -1.77083896 |
| C | 3.48275302 | -3.02019276 | -1.82966754 | H | -5.59735388 | 3.88581137 | -2.83333569 |
| H | 2.17248715 | -3.53177259 | -3.42800372 | | | | |

absolute energy *E* (B3LYP): -1829.46995232 au

[a]Calculated by DFT method [B3LYP/6-31G(d)]

**Table S6**. Coordinates (Å) and Absolute Energy of the Optimized Structure for (*P*)-**4a**

| atom | x | y | z | atom | x | y | z |
|---|---|---|---|---|---|---|---|
| H | 0.61154838 | 2.77722604 | 2.65741563 | H | 0.23422338 | -4.62288771 | -3.75386195 |
| C | 0.44336453 | 2.49320785 | 1.62322802 | C | 0.84206688 | -2.58158049 | -3.31576617 |
| H | 0.78858803 | 4.45858214 | 0.82962116 | C | 1.46308719 | -2.38411526 | -4.57044746 |
| C | 0.51677504 | 3.42891043 | 0.61127668 | C | 0.86533948 | -1.53757206 | -2.33938015 |
| C | -0.00000000 | 0.74702483 | -0.04315521 | C | 2.14960606 | -1.21954336 | -4.85149802 |
| C | 0.16722031 | 3.0867062 | -0.71466506 | H | 1.41459379 | -3.18533447 | -5.30440072 |
| C | 0.183198 | 1.16135002 | 1.28645593 | H | 2.6318035 | -1.08522136 | -5.8160014 |
| C | -0.21612986 | 1.74695365 | -1.04935685 | C | 2.24882232 | -0.22253683 | -3.86467626 |
| C | 0.17676927 | 4.10326086 | -1.72877578 | H | 2.82443586 | 0.67843341 | -4.05825752 |
| H | -1.73493798 | -0.3903263 | -1.89228766 | C | 1.62161208 | -0.3809922 | -2.6422312 |
| C | -0.25781322 | 3.8491721 | -2.9902554 | C | -0.00000000 | -0.00000000 | 2.26535895 |
| H | 0.54174243 | 5.09064904 | -1.45638665 | C | -1.24878131 | 0.31207445 | 3.1194558 |
| H | -0.23422338 | 4.62288771 | -3.75386195 | C | -3.54511614 | 1.03243895 | 4.59410741 |
| C | -0.84206688 | 2.58158049 | -3.31576617 | C | -1.1442662 | 1.07800513 | 4.29116254 |
| C | -1.46308719 | 2.38411526 | -4.57044746 | C | -2.52724612 | -0.07867715 | 2.69908031 |
| C | -0.86533948 | 1.53757206 | -2.33938015 | C | -3.66324399 | 0.27574502 | 3.42841977 |
| C | -2.14960606 | 1.21954336 | -4.85149802 | C | -2.27853503 | 1.43311553 | 5.0214215 |
| H | -1.41459379 | 3.18533447 | -5.30440072 | H | -2.63810979 | -0.66543128 | 1.79299008 |

| | | | | | | | |
|---|---|---|---|---|---|---|---|
| H | -2.6318035 | 1.08522136 | -5.8160014 | H | -4.64260419 | -0.0433885 | 3.08130047 |
| C | -2.24882232 | 0.22253683 | -3.86467626 | H | -2.16887536 | 2.02313633 | 5.92777576 |
| H | -2.82443586 | -0.67843341 | -4.05825752 | H | -4.4290332 | 1.30582751 | 5.16407618 |
| C | -1.62161208 | 0.3809922 | -2.6422312 | C | 1.24878131 | -0.31207445 | 3.1194558 |
| H | -0.61154838 | -2.77722604 | 2.65741563 | C | 3.54511614 | -1.03243895 | 4.59410741 |
| C | -0.44336453 | -2.49320785 | 1.62322802 | C | 2.52724612 | 0.07867715 | 2.69908031 |
| H | -0.78858803 | -4.45858214 | 0.82962116 | C | 1.1442662 | -1.07800513 | 4.29116254 |
| C | -0.51677504 | -3.42891043 | 0.61127668 | C | 2.27853503 | -1.43311553 | 5.0214215 |
| C | 0.00000000 | -0.74702483 | -0.04315521 | C | 3.66324399 | -0.27574502 | 3.42841977 |
| C | -0.16722031 | -3.0867062 | -0.71466506 | H | 2.63810979 | 0.66543128 | 1.79299008 |
| C | -0.183198 | -1.16135002 | 1.28645593 | H | 2.16887536 | -2.02313633 | 5.92777576 |
| C | 0.21612986 | -1.74695365 | -1.04935685 | H | 4.64260419 | 0.0433885 | 3.08130047 |
| C | -0.17676927 | -4.10326086 | -1.72877578 | H | 4.4290332 | -1.30582751 | 5.16407618 |
| H | 1.73493798 | 0.3903263 | -1.89228766 | H | 0.16632577 | -1.38888575 | 4.64553726 |
| C | 0.25781322 | -3.8491721 | -2.9902554 | H | -0.16632577 | 1.38888575 | 4.64553726 |
| H | -0.54174243 | -5.09064904 | -1.45638665 | | | | |
| absolute energy *E* (B3LYP): -1578.05941319 au | | | | | | | |

[a]Calculated by DFT method [B3LYP/6-31G(d)]

**Table S7**. Coordinates (Å) and Absolute Energy of the Optimized Structure for (*P*)-**4b**

| atom | x | y | z | atom | x | y | z |
|---|---|---|---|---|---|---|---|
| H | 2.53764126 | -2.28660502 | 1.65757586 | H | -1.56647155 | 4.44746355 | -2.55733769 |
| C | 1.49884737 | -2.10544477 | 1.39638407 | H | -3.87217431 | 4.3366411 | -1.68013278 |
| H | 0.71588138 | -3.76730996 | 2.50983612 | C | -3.45061899 | 2.71197263 | -0.29771002 |
| C | 0.49215168 | -2.93480678 | 1.84758763 | C | -4.71040322 | 2.78475795 | 0.33982586 |
| C | -0.18232724 | -0.6864793 | 0.29938771 | C | -2.480686 | 1.76317769 | 0.15237848 |
| C | -0.83821541 | -2.7625902 | 1.40130918 | C | -5.00235533 | 1.99654303 | 1.43535626 |
| C | 1.14884618 | -0.9958176 | 0.62391299 | H | -5.43952338 | 3.50026839 | -0.03351281 |
| C | -1.18500297 | -1.69006556 | 0.51477722 | H | -5.9706017 | 2.07065106 | 1.92297577 |
| C | -1.84712375 | -3.69100672 | 1.8284666 | C | -4.02198412 | 1.12157643 | 1.9362668 |
| H | -2.0504966 | -0.34697679 | -1.72861468 | H | -4.22445007 | 0.52960151 | 2.82456487 |
| C | -3.11334112 | -3.63608098 | 1.34007652 | C | -2.79473499 | 1.01023402 | 1.30815474 |
| H | -1.56646905 | -4.44746747 | 2.55733417 | C | 2.12342492 | 0.00000017 | 0.00000005 |
| H | -3.87217205 | -4.33664491 | 1.68012981 | C | 2.99734176 | -0.69494558 | -1.04925879 |
| C | -3.4506178 | -2.71197422 | 0.29770932 | C | 4.58366608 | -1.97048064 | -3.00125736 |
| C | -4.7104022 | -2.78475896 | -0.3398263 | C | 4.39292481 | -0.65263437 | -0.98990467 |
| C | -2.48068536 | -1.76317818 | -0.15237797 | C | 2.41477672 | -1.3949118 | -2.11750485 |
| C | -5.00235503 | -1.99654214 | -1.43535515 | C | 3.18749353 | -2.02695656 | -3.08474786 |
| H | -5.43952195 | -3.50027032 | 0.03351138 | C | 5.18615912 | -1.28392797 | -1.95580343 |
| H | -5.97060159 | -2.07064966 | -1.92297442 | H | 1.33108571 | -1.43963919 | -2.17965362 |
| C | -4.02198447 | -1.12157402 | -1.93626434 | H | 2.70659115 | -2.56120451 | -3.898821 |
| H | -4.22445107 | -0.52959742 | -2.82456114 | H | 6.26542894 | -1.21862941 | -1.86016223 |
| C | -2.79473515 | -1.01023225 | -1.30815252 | H | 5.19979348 | -2.46049015 | -3.74999788 |
| H | 2.53763987 | 2.28660411 | -1.65757779 | C | 2.99734101 | 0.69494653 | 1.0492591 |
| C | 1.49884614 | 2.10544379 | -1.39638538 | C | 4.58366394 | 1.97048266 | 3.00125809 |
| H | 0.71587912 | 3.76730739 | -2.50983905 | C | 2.41477521 | 1.39491239 | 2.11750498 |
| C | 0.49214999 | 2.93480488 | -1.84758952 | C | 4.3929241 | 0.65263622 | 0.98990538 |
| C | -0.18232759 | 0.68647877 | -0.29938708 | C | 5.18615772 | 1.28393036 | 1.95580435 |
| C | -0.83821691 | 2.76258821 | -1.40131052 | C | 3.18749133 | 2.02695768 | 3.08474819 |
| C | 1.14884561 | 0.99581719 | -0.6239132 | H | 1.33108415 | 1.43963907 | 2.17965344 |
| C | -1.18500378 | 1.69006453 | -0.5147771 | H | 6.26542762 | 1.21863249 | 1.86016346 |

| | | | | | | | |
|---|---|---|---|---|---|---|---|
| C | -1.84712574 | 3.69100367 | -1.82846903 | H | 2.70658837 | 2.56120533 | 3.89882118 |
| H | -2.05049592 | 0.34697999 | 1.72861813 | H | 5.19979081 | 2.46049258 | 3.74999878 |
| C | -3.113343 | 3.63607797 | -1.34007868 | O | 5.08614686 | 0.00000114 | 0.00000046 |
| absolute energy *E* (B3LYP): -1652.08619966 au | | | | | | | |

[a]Calculated by DFT method [B3LYP/6-31G(d)]

**Table S8**. Coordinates (Å) and Absolute Energy of the Optimized Structure for (*P*)-**5a**

| atom | x | y | z | atom | x | y | z |
|---|---|---|---|---|---|---|---|
| H | 0.3585589 | 2.69633619 | 3.22849199 | C | -0.03958533 | -1.1237271 | 1.76530482 |
| C | 0.2235869 | 2.45426253 | 2.17985451 | C | 0.28013511 | -1.77154257 | -0.56471839 |
| H | 0.46936936 | 4.45487591 | 1.47092122 | C | 0.02548857 | -4.17317494 | -1.12034011 |
| C | 0.27227656 | 3.41996298 | 1.2036004 | H | 1.62932555 | 0.4116395 | -1.53944539 |
| C | -0.04831547 | 0.73094554 | 0.40417184 | C | 0.39606031 | -3.95364603 | -2.41085538 |
| C | 0.00000000 | 3.11270126 | -0.15676138 | H | -0.26152186 | -5.16872099 | -0.78968394 |
| C | 0.03958533 | 1.1237271 | 1.76530482 | H | 0.39607144 | -4.76328696 | -3.13653942 |
| C | -0.28013511 | 1.77154257 | -0.56471839 | C | 0.88142734 | -2.66922807 | -2.81240325 |
| C | -0.02548857 | 4.17317494 | -1.12034011 | C | 1.44202567 | -2.48257562 | -4.0980968 |
| H | -1.62932555 | -0.4116395 | -1.53944539 | C | 0.87001352 | -1.58271817 | -1.88129646 |
| C | -0.39606031 | 3.95364603 | -2.41085538 | C | 2.0415412 | -1.28996593 | -4.44895862 |
| H | 0.26152186 | 5.16872099 | -0.78968394 | H | 1.41899628 | -3.31431212 | -4.79870919 |
| H | -0.39607144 | 4.76328696 | -3.13653942 | H | 2.4787169 | -1.16514659 | -5.4360578 |
| C | -0.88142734 | 2.66922807 | -2.81240325 | C | 2.11469151 | -0.24802393 | -3.50539061 |
| C | -1.44202567 | 2.48257562 | -4.0980968 | H | 2.62599055 | 0.67729563 | -3.75606721 |
| C | -0.87001352 | 1.58271817 | -1.88129646 | C | 1.54047208 | -0.39326143 | -2.25680405 |
| C | -2.0415412 | 1.28996593 | -4.44895862 | N | -0.00000000 | 0.00000000 | 2.57929658 |
| H | -1.41899628 | 3.31431212 | -4.79870919 | C | 0.00000000 | 0.00000000 | 4.0030895 |
| H | -2.4787169 | 1.16514659 | -5.4360578 | C | 0.00000000 | 0.00000000 | 6.79868842 |
| C | -2.11469151 | 0.24802393 | -3.50539061 | C | 0.99861914 | -0.68746101 | 4.70343343 |
| H | -2.62599055 | -0.67729563 | -3.75606721 | C | -0.99861914 | 0.68746101 | 4.70343343 |
| C | -1.54047208 | 0.39326143 | -2.25680405 | C | -0.98969283 | 0.69251633 | 6.09816924 |
| H | -0.3585589 | -2.69633619 | 3.22849199 | C | 0.98969283 | -0.69251633 | 6.09816924 |
| C | -0.2235869 | -2.45426253 | 2.17985451 | H | 1.77714712 | -1.20456084 | 4.15124276 |
| H | -0.46936936 | -4.45487591 | 1.47092122 | H | -1.77714712 | 1.20456084 | 4.15124276 |
| C | -0.27227656 | -3.41996298 | 1.2036004 | H | -1.76575491 | 1.22894238 | 6.63701096 |
| C | 0.04831547 | -0.73094554 | 0.40417184 | H | 1.76575491 | -1.22894238 | 6.63701096 |
| C | -0.00000000 | -3.11270126 | -0.15676138 | H | -0.00000000 | 0.00000000 | 7.88501825 |
| absolute energy *E* (B3LYP): -1363.07317530 au | | | | | | | |

[a]Calculated by DFT method [B3LYP/6-31G(d)]

**Table S9**. Coordinates (Å) and Absolute Energy of the Optimized Structure for (*P*)-**5b**

| atom | x | y | z | atom | x | y | z |
|---|---|---|---|---|---|---|---|
| H | 2.94557725 | -2.58896837 | 0.8186557 | C | -1.40833552 | 4.11434072 | -0.70008642 |
| C | 1.89629279 | -2.37564121 | 0.64541176 | H | -1.825751 | -0.12252332 | 1.67502346 |
| H | 1.18981888 | -4.30443776 | 1.23410745 | C | -2.69874427 | 3.96230313 | -0.29662573 |
| C | 0.92130624 | -3.31944283 | 0.86116343 | H | -1.07807127 | 5.04499861 | -1.15578411 |
| C | 0.11821905 | -0.7273846 | 0.07972093 | H | -3.42463769 | 4.75949954 | -0.43703996 |
| C | -0.43964864 | -3.06539935 | 0.54071502 | C | -3.09977867 | 2.78163334 | 0.4045583 |
| C | 1.4799734 | -1.09742837 | 0.23446689 | C | -4.38526396 | 2.69478482 | 0.98951503 |
| C | -0.84940034 | -1.79358031 | 0.03248169 | C | -2.16838146 | 1.70982685 | 0.58156389 |
| C | -1.40196684 | -4.11526168 | 0.70003646 | C | -4.73558739 | 1.62425903 | 1.78707386 |

| | | | | | | | |
|---|---|---|---|---|---|---|---|
| H | -1.82701514 | 0.12118111 | -1.6744264 | H | -5.0861366 | 3.50974061 | 0.82271135 |
| C | -2.69287024 | -3.96501726 | 0.29749881 | H | -5.72252526 | 1.57697287 | 2.23963566 |
| H | -1.07005027 | -5.04551108 | 1.15536883 | C | -3.79170652 | 0.61101059 | 2.03956653 |
| H | -3.41752455 | -4.76327093 | 0.43830686 | H | -4.04199014 | -0.21159562 | 2.70378147 |
| C | -3.09606631 | -2.78484633 | -0.40328699 | C | -2.54338347 | 0.65462787 | 1.4483253 |
| C | -4.38205096 | -2.69980857 | -0.98740786 | N | 2.29285302 | 0.00227094 | -0.0006782 |
| C | -2.16632678 | -1.71168432 | -0.58079955 | C | 3.71691971 | 0.00270869 | -0.00104383 |
| C | -4.73442176 | -1.62974196 | -1.78468129 | C | 6.53785082 | 0.00642798 | -0.00505971 |
| H | -5.0816408 | -3.51578579 | -0.82021277 | C | 4.42222595 | 0.84377436 | 0.86651071 |
| H | -5.72171403 | -1.58384833 | -2.23661277 | C | 4.42204369 | -0.83472393 | -0.87246831 |
| C | -3.79215118 | -0.61513803 | -2.03773031 | C | 5.81555599 | -0.83339965 | -0.86351347 |
| H | -4.04402752 | 0.20713442 | -2.70175575 | C | 5.81579524 | 0.84801676 | 0.85171375 |
| C | -2.5433916 | -0.65699272 | -1.44728113 | H | 3.87574203 | 1.48691375 | 1.54948321 |
| H | 2.94128084 | 2.59427343 | -0.82163807 | H | 3.87518796 | -1.47318263 | -1.55952026 |
| C | 1.89246393 | 2.37946668 | -0.64740978 | H | 6.35109925 | -1.4879658 | -1.54714645 |
| H | 1.18279424 | 4.3070198 | -1.23627544 | H | 6.35138183 | 1.5108892 | 1.52723054 |
| C | 0.91595395 | 3.32177467 | -0.86279626 | C | 8.04830224 | -0.0132784 | 0.01358581 |
| C | 0.11715638 | 0.72876808 | -0.08024977 | H | 8.45780769 | -0.27845766 | -0.96680713 |
| C | -0.44440573 | 3.06588229 | -0.54128594 | H | 8.42666968 | -0.75058209 | 0.73421862 |
| C | 1.4782984 | 1.10076561 | -0.23575393 | H | 8.45874693 | 0.96058085 | 0.30025303 |
| C | -0.85197503 | 1.79356949 | -0.03256284 | | | | |

absolute energy *E* (B3LYP): -1402.39144844 au

[a]Calculated by DFT method [B3LYP/6-31G(d)]

**Table S10**. Coordinates (Å) and Absolute Energy of the Optimized Structure for (*P*)-**5c**

| atom | x | y | z | atom | x | y | z |
|---|---|---|---|---|---|---|---|
| H | 2.62650289 | -2.67115348 | 0.74930426 | C | -1.59600423 | 4.12518513 | -0.71845005 |
| C | 1.57857993 | -2.43572299 | 0.59686354 | H | -2.07563893 | -0.09093886 | 1.68112825 |
| H | 0.84060385 | -4.34739184 | 1.20339683 | C | -2.88254917 | 4.00663466 | -0.29202235 |
| C | 0.58723872 | -3.35727974 | 0.83336301 | H | -1.2509745 | 5.04568603 | -1.18380933 |
| C | -0.17356015 | -0.74961299 | 0.05828941 | H | -3.59102293 | 4.82091702 | -0.4231759 |
| C | -0.77365823 | -3.07349604 | 0.53821857 | C | -3.30005882 | 2.83893544 | 0.42111633 |
| C | 1.18229386 | -1.1496174 | 0.19148625 | C | -4.57674773 | 2.78597411 | 1.0289101 |
| C | -1.16493985 | -1.7944308 | 0.03311136 | C | -2.3922622 | 1.74519167 | 0.58628627 |
| C | -1.75577148 | -4.10123385 | 0.7193622 | C | -4.93915696 | 1.72751966 | 1.83713017 |
| H | -2.13205589 | 0.13513794 | -1.66268038 | H | -5.26026929 | 3.61726572 | 0.87080183 |
| C | -3.05047133 | -3.92402695 | 0.34064244 | H | -5.91887505 | 1.70629168 | 2.30703954 |
| H | -1.43577132 | -5.03712381 | 1.17170939 | C | -4.01615576 | 0.69232525 | 2.07734776 |
| H | -3.78967442 | -4.70571223 | 0.49791643 | H | -4.27481745 | -0.12138265 | 2.74928237 |
| C | -3.44075809 | -2.73766381 | -0.35693954 | C | -2.77779809 | 0.70288822 | 1.46398425 |
| C | -4.73530462 | -2.62612387 | -0.91717402 | N | 2.0154028 | -0.0713869 | -0.06560173 |
| C | -2.49106192 | -1.68594125 | -0.55593302 | C | 3.43962876 | -0.10450565 | -0.09105457 |
| C | -5.07896016 | -1.55128084 | -1.711848 | C | 6.24283381 | -0.16776371 | -0.14258018 |
| H | -5.44945366 | -3.42585965 | -0.73372071 | C | 4.1802612 | 0.66786982 | 0.80517148 |
| H | -6.07336147 | -1.48512701 | -2.14532326 | C | 4.11247274 | -0.91365035 | -1.01885025 |
| C | -4.11948525 | -0.55866766 | -1.98641369 | C | 5.49948038 | -0.95229894 | -1.03882154 |
| H | -4.36556526 | 0.26671556 | -2.64876171 | C | 5.57643683 | 0.64922303 | 0.77934086 |
| C | -2.86106728 | -0.62617471 | -1.41925839 | H | 3.66134374 | 1.28567179 | 1.53173925 |
| H | 2.71314955 | 2.49812664 | -0.90657119 | H | 3.53886672 | -1.5064013 | -1.72476093 |
| C | 1.6619239 | 2.31063996 | -0.71540933 | H | 6.03371275 | -1.57462182 | -1.74953287 |

| | | | | | | | |
|---|---|---|---|---|---|---|---|
| H | 0.98927907 | 4.2535228 | -1.29848977 | H | 6.1265079 | 1.26112288 | 1.48464058 |
| C | 0.7052489 | 3.27600545 | -0.91736128 | O | 7.59665813 | -0.27154718 | -0.25088947 |
| C | -0.14365889 | 0.70519035 | -0.11152715 | C | 8.40505375 | 0.49788213 | 0.6271558 |
| C | -0.65534986 | 3.05405606 | -0.57210318 | H | 8.2325997 | 1.57395643 | 0.49446017 |
| C | 1.22320584 | 1.04362153 | -0.29396227 | H | 9.43777125 | 0.26294282 | 0.36429034 |
| C | -1.08517417 | 1.79379907 | -0.05130942 | H | 8.22783186 | 0.2297412 | 1.67687515 |

absolute energy *E* (B3LYP): -1477.59605286 au

[a]Calculated by DFT method [B3LYP/6-31G(d)]

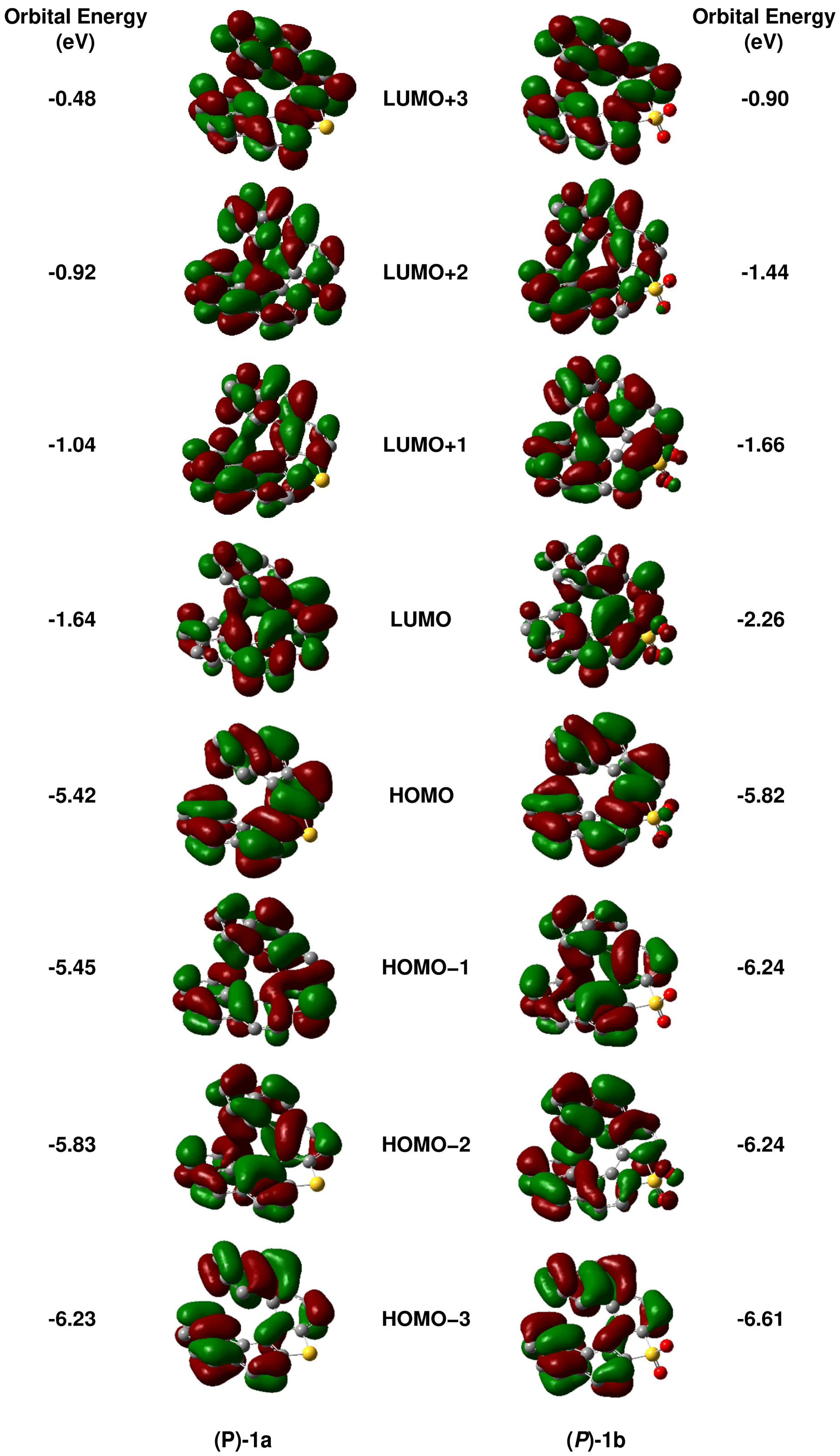


**Figure S9**. Molecular orbitals of (*P*)-**1a** and (*P*)-**1b** calculated by DFT method at the B3LYP/6-31G(d) level of theory.

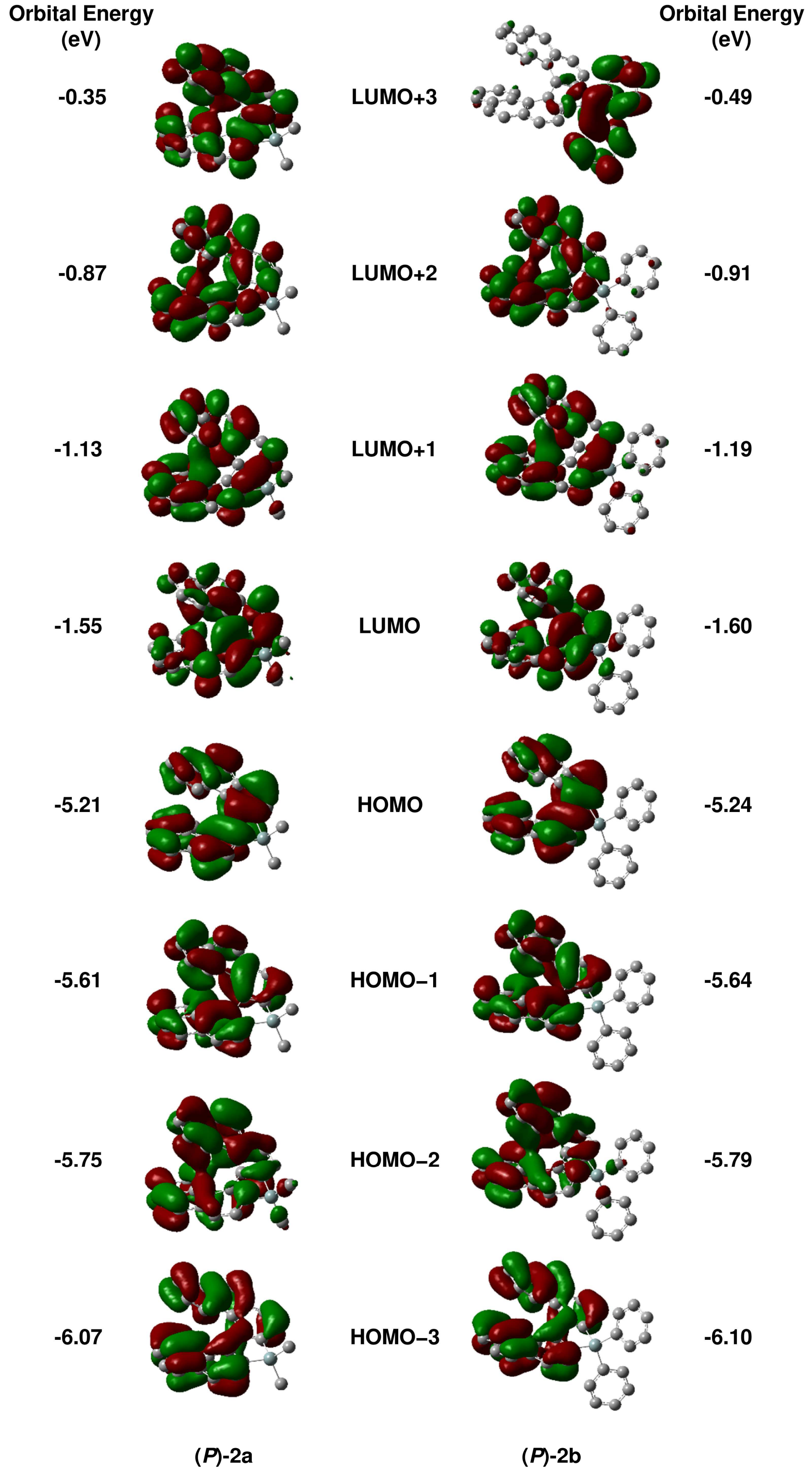


**Figure S10**. Molecular orbitals of (*P*)-**2a** and (*P*)-**2b** calculated by DFT method at the B3LYP/6-31G(d) level of theory.

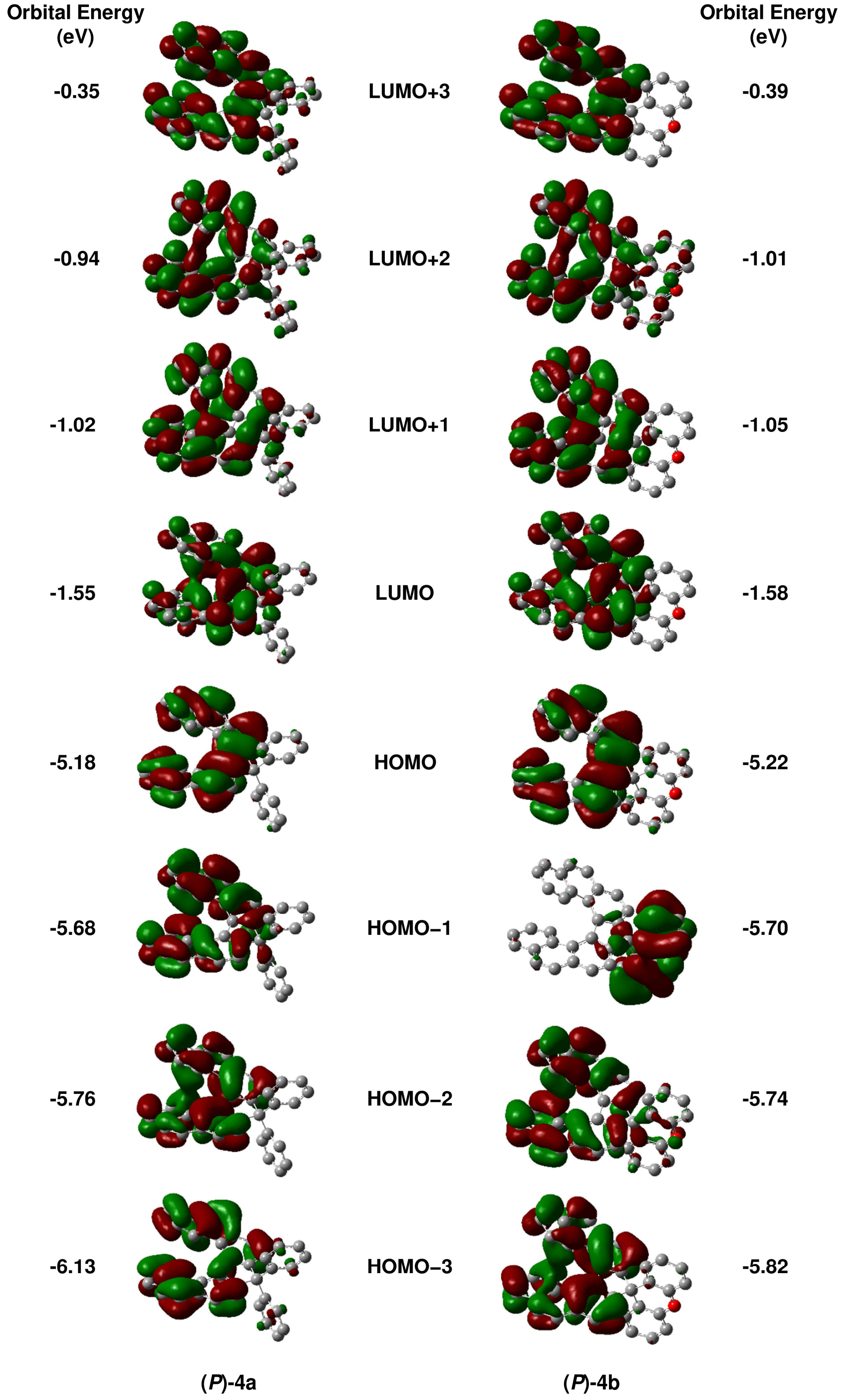


**Figure S11**. Molecular orbitals of (*P*)-**4a** and (*P*)-**4b** calculated by DFT method at the B3LYP/6-31G(d) level of theory.

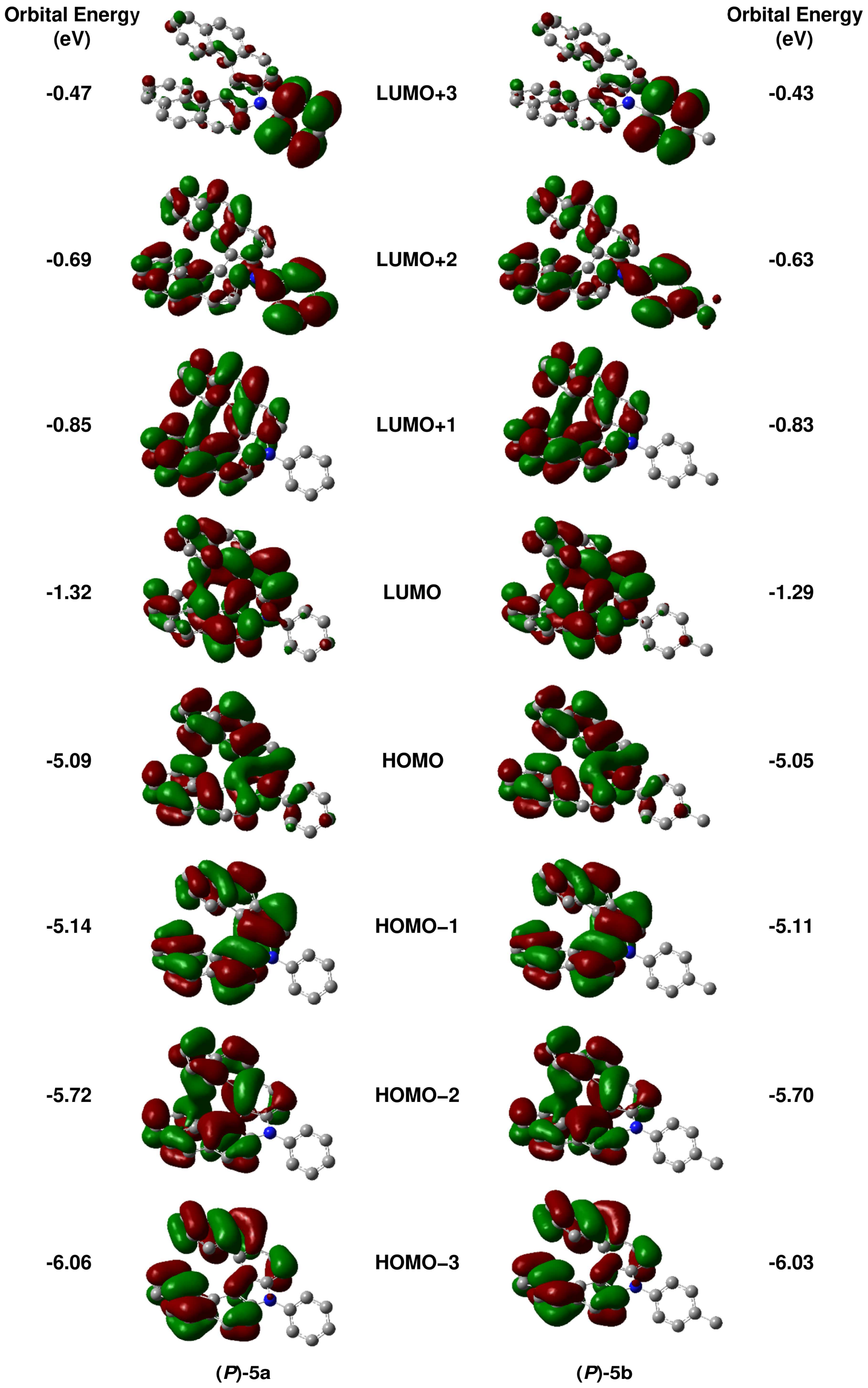


**Figure S12**. Molecular orbitals of (*P*)-**5a** and (*P*)-**5b** calculated by DFT method at the B3LYP/6-31G(d) level of theory.

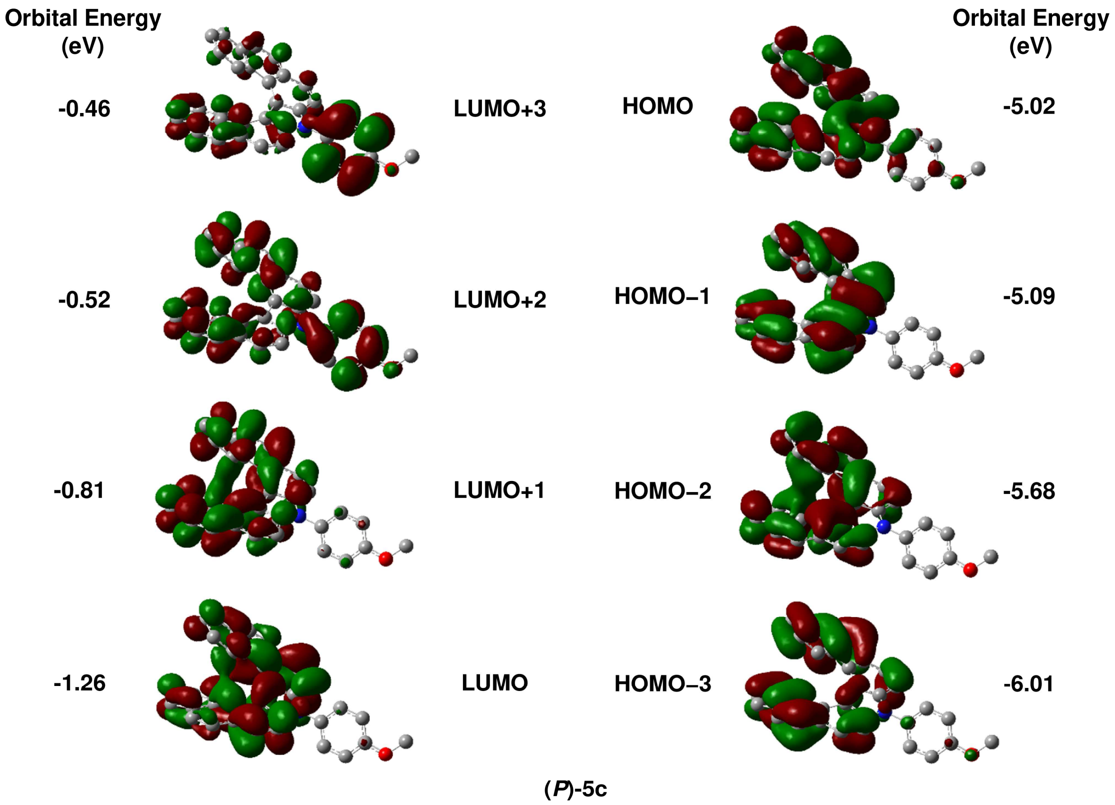


**Figure S13**. Molecular orbitals of (*P*)-**5c** calculated by DFT method at the B3LYP/6-31G(d) level of theory.

**Table S11.** The Selected Absorption of (*P*)-**1**, **2**, **4**, and **5** Calculated by TD–DFT Method at the B3LYP15/6-31G(d) Level of Theory

| | excited state | transition energy (eV) | wavelength (nm) | main transition configuration (CI expansion coefficient) | oscillator strength | Rotatory Strength ($10^{-40}$ erg· esu· cm/gauss) | | transition electric dipole moments (a.u.) | | | transition magnetic dipole moments (a.u.) | | |
|---|---|---|---|---|---|---|---|---|---|---|---|---|---|
| | | | | | *f* | $R_{velocity}$ | $R_{length}$ | x | y | z | x | y | z |
| (*P*)-**1a** | 1 | 3.207 | 387 | HOMO-1 → LUMO (0.66493) | 0.0251 | -31.9744 | -33.6058 | 0.0000 | -0.5657 | -0.0000 | 0.0000 | -0.252 | 0.0000 |
| | 2 | 3.262 | 380 | HOMO → LUMO (0.67382) | 0.1264 | 74.2516 | 67.9571 | 1.2434 | 0.0000 | -0.1888 | -0.0483 | 0.0000 | 1.2083 |
| | 3 | 3.539 | 350 | HOMO-2 → LUMO (0.67854) | 0.0331 | 216.7266 | 205.6507 | -0.4739 | -0.0000 | 0.3964 | 0.3625 | 0.0000 | -1.7674 |
| | 4 | 3.881 | 320 | HOMO-1 →LUMO+1 (0.68263) | 0.1434 | 288.3835 | 276.0247 | 1.1659 | 0.0000 | -0.3855 | -0.247 | 0.0000 | 2.2904 |
| | 5 | 3.907 | 317 | HOMO-3 → LUMO (0.54000) | 0.0094 | -12.8769 | -12.7982 | 0.0000 | 0.3142 | 0.0000 | -0.0000 | 0.1728 | 0.0000 |
| | 6 | 3.931 | 315 | HOMO → LUMO+1 (0.52264) | 0.0303 | -70.1043 | -73.6564 | -0.0000 | 0.5611 | 0.0000 | -0.0000 | 0.5569 | -0.0001 |
| (*P*)-**1b** | 1 | 3.053 | 406 | HOMO → LUMO (0.68689) | 0.0714 | -53.5581 | -50.6415 | 0.2033 | 0.9555 | -0.0000 | -0.5572 | 0.3434 | -0.0000 |
| | 2 | 3.289 | 377 | HOMO-1 → LUMO (0.62341) | 0.0373 | 49.0012 | 47.0378 | 0.1549 | -0.6625 | 0.0000 | -0.683 | 0.1415 | -0.0000 |
| | 3 | 3.388 | 366 | HOMO-2 → LUMO (0.66097) | 0.0319 | -48.3505 | -50.9174 | -0.0000 | 0.0000 | 0.6204 | 0.0000 | -0.0000 | 0.3482 |
| | 4 | 3.624 | 342 | HOMO → LUMO+1 (0.61182) | 0.1261 | 627.573 | 609.6764 | 0.6797 | -0.9787 | 0.0000 | -2.8649 | 0.6531 | -0.0000 |
| | 5 | 3.721 | 333 | HOMO-3 → LUMO (0.65739) | 0.0000 | 0.0071 | 0.0370 | 0.0000 | -0.0000 | 0.0157 | -0.0000 | 0.0000 | -0.0100 |
| | 6 | 3.962 | 313 | HOMO → LUMO+2 (0.62412) | 0.0862 | -144.5649 | -150.0925 | -0.0000 | -0.0000 | 0.9421 | 0.0000 | -0.0000 | 0.6759 |
| (*P*)-**2a** | 1 | 3.162 | 392 | HOMO → LUMO (0.68088) | 0.1156 | -20.3688 | -20.7191 | -0.038 | -1.2211 | 0.0000 | -0.4352 | -0.0584 | -0.0000 |
| | 2 | 3.307 | 375 | HOMO-1 → LUMO (0.54443) | 0.0068 | 3.1315 | 3.3418 | -0.0239 | 0.2884 | 0.0000 | 0.1728 | -0.0349 | -0.0000 |
| | 3 | 3.524 | 352 | HOMO-2 → LUMO (0.54443) | 0.0092 | -10.2421 | -10.8918 | 0.0000 | -0.0000 | 0.3261 | -0.0000 | 0.0000 | 0.1417 |
| | 4 | 3.589 | 345 | HOMO → LUMO+1 (0.52768) | 0.0802 | 700.4543 | 671.1434 | -0.8361 | 0.4612 | 0.0000 | 2.8895 | -0.9351 | -0.000 |
| | 5 | 3.848 | 322 | HOMO → LUMO+2 (0.45264) | 0.0287 | -61.9701 | -62.9384 | -0.0000 | -0.0000 | 0.5516 | -0.0000 | 0.0000 | 0.4841 |
| | 6 | 3.895 | 318 | HOMO-3 → LUMO (0.47198) | 0.0357 | -67.9232 | -74.5527 | -0.0000 | 0.0000 | 0.6115 | 0.0000 | -0.0000 | 0.5172 |
| (*P*)-**2b** | 1 | 3.133 | 396 | HOMO → LUMO (0.68486) | 0.0982 | -17.3398 | -16.2309 | 0.0000 | -0.9448 | -0.6215 | -0.0000 | -0.1638 | 0.1382 |
| | 2 | 3.276 | 378 | HOMO-1 → LUMO (0.53879) | 0.0041 | 1.9332 | 1.6765 | 0.0000 | -0.1893 | -0.1210 | -0.0000 | 0.0196 | 0.0281 |
| | 3 | 3.510 | 353 | HOMO-2 → LUMO (0.60686) | 0.0112 | -13.0184 | -13.7499 | -0.3609 | -0.0001 | 0.0000 | -0.1616 | 0.0003 | -0.0002 |
| | 4 | 3.553 | 349 | HOMO → LUMO+1 (0.52515) | 0.0929 | 752.101 | 721.54 | 0.0001 | -0.9251 | 0.4592 | -0.0000 | 2.3756 | -1.8800 |
| | 5 | 3.824 | 324 | HOMO-3 → LUMO (0.46782) | 0.0330 | -60.8655 | -61.6044 | 0.5933 | 0.0000 | -0.0000 | 0.4405 | -0.0000 | 0.0001 |
| | 6 | 3.872 | 320 | HOMO → LUMO+2 (0.45741) | 0.0586 | -95.2519 | -102.9361 | 0.7859 | -0.0000 | 0.0000 | 0.5556 | 0.0000 | 0.0000 |
| (*P*)-**4a** | 1 | 3.157 | 393 | HOMO → LUMO (0.68058) | 0.1447 | -85.522 | -82.1775 | -0.2756 | 1.3399 | 0.0000 | 0.0016 | 0.2605 | 0.0000 |
| | 2 | 3.470 | 357 | HOMO-1 → LUMO (0.59219) | 0.0073 | -12.6497 | -13.3185 | -0.0000 | 0.0000 | 0.2926 | -0.0000 | 0.0000 | 0.1931 |
| | 3 | 3.478 | 356 | HOMO-2 → LUMO (0.49445) | 0.0191 | -3.6594 | -3.6544 | -0.1052 | 0.4622 | -0.0000 | -0.0131 | 0.0306 | 0.0000 |
| | 4 | 3.724 | 333 | HOMO-2 → LUMO (0.49463) | 0.0587 | 542.469 | 521.6248 | -0.642 | 0.4803 | 0.0000 | 2.9145 | -0.7116 | -0.0000 |
| | 5 | 3.837 | 323 | HOMO → LUMO+2 (0.56369) | 0.1570 | -202.3037 | -207.0602 | 0.0000 | 0.0000 | -1.2922 | -0.0000 | -0.0000 | -0.6798 |
| | 6 | 3.974 | 312 | HOMO-3 → LUMO (0.60105) | 0.0567 | -73.1346 | -77.3123 | 0.0000 | -0.0000 | 0.763 | 0.0000 | -0.0000 | 0.4299 |

| | | | | | | | | | | | | | |
|---|---|---|---|---|---|---|---|---|---|---|---|---|---|
| (*P*)-**4b** | 1 | 3.171 | 391 | HOMO → LUMO (0.67747) | 0.1447 | -39.0831 | -37.1657 | -0.0000 | -1.3159 | 0.3612 | 0.0000 | -0.1020 | 0.0647 |
| | 2 | 3.484 | 356 | HOMO-2 → LUMO (0.54038) | 0.0009 | -3.4782 | -3.7915 | 0.103 | -0.0000 | 0.0000 | 0.1561 | -0.0000 | -0.0000 |
| | 3 | 3.504 | 354 | HOMO → LUMO+1 (0.48680) | 0.0205 | -1.5381 | -1.3386 | -0.0000 | -0.4589 | 0.1666 | -0.0000 | -0.0646 | -0.1438 |
| | 4 | 3.557 | 349 | HOMO-1 → LUMO (0.66521) | 0.0056 | -2.1916 | -2.1224 | 0.2533 | 0.0000 | 0.0000 | 0.0355 | -0.0000 | -0.0000 |
| | 5 | 3.748 | 331 | HOMO-3 → LUMO (0.51075) | 0.0678 | 575.6832 | 557.8912 | 0.0000 | -0.7367 | -0.4429 | -0.0000 | 1.7792 | 2.3842 |
| | 6 | 3.825 | 324 | HOMO → LUMO+2 (0.56366) | 0.1836 | -230.1393 | -236.992 | -1.3997 | -0.0000 | -0.0000 | -0.7183 | -0.0000 | -0.0000 |
| (*P*)-**5a** | 1 | 3.187 | 389 | HOMO → LUMO (0.66109) | 0.0409 | -51.4961 | -52.9864 | -0.0000 | -0.0000 | -0.7238 | 0.0000 | 0.0000 | -0.3105 |
| | 2 | 3.296 | 376 | HOMO-1 → LUMO (0.64999) | 0.1036 | 46.7076 | 47.6421 | 0.2548 | -1.1038 | -0.0000 | -0.7605 | 0.0076 | -0.0000 |
| | 3 | 3.760 | 330 | HOMO-2 → LUMO (0.53834) | 0.1176 | 316.6039 | 302.8194 | -0.5679 | 0.977 | 0.0000 | 2.0191 | -0.1412 | 0.0000 |
| | 4 | 3.780 | 328 | HOMO → LUMO+1 (0.59701) | 0.1755 | 197.6476 | 196.6378 | -0.4782 | 1.291 | 0.0000 | 1.7426 | -0.0006 | -0.0000 |
| | 5 | 3.843 | 323 | HOMO → LUMO+2 (0.63485) | 0.0217 | 13.1795 | 12.3393 | -0.0000 | 0.0000 | -0.4803 | 0.0000 | 0.0000 | 0.109 |
| | 6 | 3.882 | 319 | HOMO-1 →LUMO+1 (0.58640) | 0.2091 | -209.9141 | -213.9548 | 0.0000 | -0.0000 | -1.4828 | -0.0000 | -0.0000 | -0.6121 |
| (*P*)-**5b** | 1 | 3.184 | 389 | HOMO → LUMO (0.66146) | 0.0425 | -52.1464 | -53.761 | -0.7385 | -0.0003 | -0.0003 | -0.3088 | -0.002 | -0.0011 |
| | 2 | 3.295 | 376 | HOMO-1 → LUMO (0.64832) | 0.1008 | 45.5397 | 46.3121 | -0.0014 | 1.1159 | 0.0592 | -0.0002 | -0.1408 | -0.6643 |
| | 3 | 3.765 | 329 | HOMO → LUMO+1 (0.56690) | 0.2748 | 549.7986 | 534.2344 | -0.0002 | -1.6587 | -0.4771 | 0.0013 | 0.6369 | 2.5363 |
| | 4 | 3.779 | 328 | HOMO-2 → LUMO (0.52395) | 0.0246 | -8.2769 | -7.5477 | 0.0018 | 0.515 | -0.0315 | 0.0006 | 0.0499 | -0.2002 |
| | 5 | 3.854 | 322 | HOMO → LUMO+2 (0.47594) | 0.0030 | -11.0967 | -14.602 | 0.1779 | 0.0055 | 0.0005 | 0.3485 | -0.0062 | -0.0102 |
| | 6 | 3.892 | 319 | HOMO → LUMO+2 (0.46655) | 0.2407 | -192.0264 | -193.456 | -1.5888 | 0.0035 | -0.0021 | -0.5166 | -0.0088 | -0.0042 |
| (*P*)-**5c** | 1 | 3.183 | 389 | HOMO → LUMO (0.66137) | 0.0393 | -49.4711 | -51.0653 | -0.7097 | 0.0131 | 0.0063 | -0.3052 | -0.0076 | 0.0171 |
| | 2 | 3.297 | 376 | HOMO-1 → LUMO (0.64605) | 0.0973 | 43.8398 | 44.3407 | 0.0246 | 1.0961 | 0.0512 | -0.0213 | -0.1439 | -0.5834 |
| | 3 | 3.760 | 330 | HOMO → LUMO+1 (0.65072) | 0.2990 | 498.114 | 488.0515 | -0.0577 | -1.7486 | -0.4305 | 0.0651 | 0.6082 | 2.3306 |
| | 4 | 3.797 | 327 | HOMO-2 → LUMO (0.63461) | 0.0113 | 88.6109 | 82.6567 | -0.0034 | -0.2279 | -0.2632 | 0.0302 | 0.3837 | 0.9998 |
| | 5 | 3.857 | 321 | HOMO-1 →LUMO+1 (0.60073) | 0.0705 | -101.0121 | -106.7926 | -0.8632 | 0.0164 | 0.0156 | -0.5242 | 0.0125 | 0.0206 |
| | 6 | 3.943 | 314 | HOMO → LUMO+2 (0.56263) | 0.1081 | -75.6532 | -75.8825 | -1.0573 | 0.0051 | -0.0393 | -0.31 | -0.0878 | 0.1372 |
